# Socioeconomic circumstances, ethnicity, migration and unintentional early childhood injuries: an analysis of the UK millennium cohort study

**DOI:** 10.1186/s40621-025-00603-y

**Published:** 2025-09-02

**Authors:** Laura Gallagher, Emma Curran, Michael Rosato, Gerard Leavey

**Affiliations:** Bamford Centre for Mental Health and Wellbeing, Ulster University, Northern Ireland, BT52 1SA UK

**Keywords:** Unintentional injury, Accidental injuries, Injury prevention, Millennium cohort study, Ethnic group, Child, Minority groups, Parent

## Abstract

**Background:**

Although health inequalities associated with ethnic disadvantage are of increasing concern to policymakers in the United Kingdom (UK), evidence on ethnicity and childhood unintentional injuries is unclear. Given that people from some minority ethnic communities face disproportionate disadvantage such as unemployment, poverty, and insecure and low-quality housing, children from these families might be expected to have higher risks of unintentional injuries compared to their White counterparts.

**Aims:**

To determine whether the likelihood of unintentional childhood injuries vary among children from minority ethnic backgrounds and whether this variation can be explained by maternal migration status and variables relating to household composition, parenting attitudes and behaviours.

**Methods:**

We used logistic regression to analyse data from 12,717 children using sweeps two (2003–2004) and three (2005–2006) of the Millennium Cohort Study. Unintentional childhood injuries were measured in the third sweep of data collection when the children were aged five. Exposure variables included socioeconomic information, ethnicity, housing, household composition, maternal migration status and variables relating to parenting, values, and behaviours.

**Results:**

Children from some minority ethnic backgrounds (Pakistani, Bangladeshi, Black African, and ‘other’) were less likely to be injured than White children. Having a mother who was born outside the UK explained the relationship in Pakistani and Bangladeshi children. We observed differences in variables such as parenting style, values, household composition, and smoking and alcohol use among minority ethnic and migrant groups, but these variables did not statistically explain the differences in childhood injury.

**Conclusions:**

Children from minority ethnic families in the UK are less likely to sustain unintentional injuries compared to their White peers, with this protective effect primarily evident among children whose mothers were born outside the UK. While cultural and behavioural differences were observed between ethnic groups, these did not statistically explain the injury variation. The findings emphasise the importance of disaggregating ethnicity and migrant status in injury prevention research and investigating the mechanisms underlying lower injury rates among first-generation migrant families.

**Supplementary Information:**

The online version contains supplementary material available at 10.1186/s40621-025-00603-y.

## Background

Childhood injuries can be classified as intentional or unintentional, and by causal mechanism. Thus, intentional injuries include those from assault, self-harm and suicide, and war; those classified as unintentional include road traffic injuries, burns, sports injuries, falls, drowning, suffocation, and poisonings. Most childhood injuries are unintentional and are a persistent public health concern, bringing an estimated 7% of deaths in children under five in England and result in disability and loss of quality of life [1].

Socioeconomic risk factors for childhood injury include social deprivation and living in social housing [2]. Thus, the immediate household environment for poor families tend to have deteriorated structures and be more exposed to hazardous utilities [3]. The fundamental causes theory suggests that various resources required for the protection of an individual’s (and their family’s) health and safety are socially distributed and differentially available [4]. People of higher socioeconomic status are afforded better health outcomes because of their access to prevention and treatment measures. Poverty and material deprivation are acknowledged risk factors of child injuries, not just because poor families living in deprived areas are more exposed to a wider range of hazards, a “proximate tier” but are also without access to the means to protect themselves at home or in the community [6]. These deficits can also be attributed to Bourdieu’s concepts of lower educational capital (knowledge of potential hazard) and lower social capital (poor social networks) [7].

Despite being disproportionately represented in lower socioeconomic strata and living in the most insecure and poor-quality housing [8, 9], there is some evidence that minority ethnic children in the UK are at lower risk of unintentional injuries compared to their White counterparts [10]. Black and South Asian children have a lower fracture incidence [11] and South Asian children are at lower risk of needing hospital care for unintentional injury [12]. Previous studies have asserted that higher bone density in Black children may explain their lower fracture incidence and lower physical activity explains fewer fractures among South Asian children [11]. However, a Dutch study using the Generation R birth cohort found that children of European ancestry were twice as likely as children of Asian ancestry, and 1.5 times as likely as children of African ancestry to sustain a fracture after controlling for both physical activity and bone density [13]. Biological explanations fail to account for lower fracture risk and no study has investigated structural and cultural factors which may explain lower injury risk among minority ethnic children in the UK.

In the UK, many migrants report good health upon arrival, but this often declines over time [14] and this phenomenon has also been observed for unintentional childhood injuries [15]. Lower unintentional injury among minority ethnicity children may also be due to a mixture of cultural and structural differences such as multigenerational living, values in parenting and childrearing, regular routines, or strict discipline [16]. Grandparental co-residence has been associated with fewer unintentional injuries in Japanese and UK samples [17, 18]. Having more adults involved in the day-to-day care of children may increase the time they are under close proximity supervision, reducing injury risk [19]. Limitations in administrative healthcare data mean many studies are unable to control for migrant status when investigating the link between ethnicity and injury [20]. Further, studies tend to dichotomise ethnicity when controlling for ethnicity in studies where it is not the main outcome [10].

Despite evidence of lower injury rates among some minority ethnic children, current UK injury prevention policies do not adequately distinguish between ethnicity and migration status in their targeting strategies. While the National Institute for Health and Care Excellence (NICE) recommends accounting for cultural beliefs in home safety interventions, broader policy frameworks such as Public Health England’s injury prevention guidance focus primarily on socioeconomic disadvantage without considering ethnic-specific protective factors or migration-related influences [21, 22]. This represents a missed opportunity to develop more nuanced, evidence-based prevention strategies that could benefit from understanding the mechanisms underlying lower injury rates in certain communities.

We sought to (1) determine whether the likelihood of unintentional childhood injury varied among children from minority ethnic backgrounds and any association with mother’s country of origin; and (2) explore ethnic variations in parenting practices, household composition, and value systems, and the extent to which these factors reduce risk of injury. We hypothesised that children from minority ethnic backgrounds would experience fewer unintentional injuries compared to White children, consistent with previous UK research. We further hypothesised that children whose mothers were born outside the UK would experience fewer injuries and that this would explain some of the variation in injury by ethnicity.

## Methods

### Sample

The Millennium Cohort Study (MCS) is a UK longitudinal birth cohort study following 18,551 families, initiated in 2001 to explore early-life influences on life outcomes. The sample included children born between September 2000 and January 2002 in England, Wales, Scotland, and Northern Ireland [23]. This study used data from sweeps two and three when the children were aged three (2003–2004) and five (2005–2006) respectively. In the first sweep, 18,552 families were included in the survey. An additional 692 families were recruited at the sweep two survey and were eligible when the child was nine months old but were not identified by the child benefit system in time to participate. Of these, 15,590 participated in the second seep of data collection, and 15,246 participated in the third sweep. For the present study, data were restricted to singleton births with biological mothers as primary respondents and complete data (*n* = 12,717).

The MCS used a stratified, clustered sampling design, oversampling disadvantaged, and ethnically diverse areas. Stratification involved three strata in England (ethnic minority, disadvantaged, advantaged) and two in other UK regions (disadvantaged, advantaged). Ethical approval was granted by NHS (National Health Service) Research Ethics Committees, with informed parental consent collected at each stage [24]. Data were sourced from the UK Data Service in May 2020.

### Measures

The outcome variable, unintentional injury, was reported during the third sweep of data collection when the cohort members were five years old. All exposure variables and covariates were measured at the second sweep of data collection when the cohort members were three years old.

#### Injury

The main outcome variable, unintentional childhood injury, was measured at sweep three when the child was five years old. Respondents were asked if, since the last interview, which was conducted when the children were around three years old, the child “had an accident or injury for which he or she has been taken to the doctor, health centre, or hospital?”

#### Ethnic group

Ethnic group refers to the ethnic group of the child reported by the main respondent. In our study, we used the 11-category Census ethnic group variable reported during the second sweep of data collection. Questions on ethnic identity are based on categories from the UK Census and include: White, Indian, Pakistani or Bangladeshi, Black Caribbean, Black African, and other. The “other” category includes individuals of mixed ethnic heritage and those selecting ‘other ethnic group’. Combining Pakistani and Bangladeshi groups was necessary due to small numbers in these subsamples. However, they share similar socioeconomic, religious, and cultural characteristics [25].

#### Maternal country of birth

Respondents were asked to indicate whether they were born in the UK, a binary variable.

### Covariates

#### Socioeconomic, cultural, and housing variables

Poverty was defined as income below 60% of the national median. Housing tenure was categorized as: (1) own home/mortgage, (2) local authority rent, (3) housing association rent, and (4) private rent. Maternal education level was dichotomised, and any parental employment was a binary variable. Religion included five groups: no religion, Christian, Muslim, Hindu and Sikh, and other. Household composition was coded as single- or two-parent households. Housing quality, a composite measure, included crowding, dwelling type, garden access, heating, damp, and neighbourhood safety (α = 0.50).

#### Parental and supervision variables

Parental factors included maternal age (11–19, 20–29, 30–40, 40+), grandparental co-residence, presence of other adult residents, and household chaos which was measured based on three statements: ‘you can’t hear yourself think in your home,’ ‘the atmosphere in your home is calm,’ and ‘it’s really disorganized in your home.’ Additional variables included smoking in the home, maternal alcohol use, and number of siblings.

Maternal social capital was composed of time spent with friends, contact with parents, perception of the local area as a place to raise children, and overall satisfaction with the local area (α = 0.29).

#### Values related to family and parenting

Respondents rated the following values: independence, obedience and respect, negotiation skills, respect for elders, academics, and religious values. Maternal attitude toward working mothers was assessed using a five-point Likert scale based on the statement: ‘A child is likely to suffer if his or her mother works before starting school.’ This variable was dichotomized into (1) positive or (2) neutral or negative attitudes toward working mothers. Additional variables included parenting style (structured vs. unstructured), allowance of screen time, and adherence to a regular bedtime.

### Analyses and model specification

Analyses were conducted using complete cases, with weighting variables applied to account for stratification and clustering. Model specification was carried out based on Hosmer et al.’s process [26]. Variables were selected based on their association with unintentional childhood injury and ethnicity. Bivariate analyses were performed to assess the relationship between individual candidate variables and the outcome which was injury count at age five. A commonly cited criteria for the inclusion of a variable in the multivariate model is a bivariate association with the outcome at a significance of *p* < 0.25. The next step was to eliminate variables which displayed collinearity. We further removed variables which resulted in significant data sparsity including the variables relating to values and maternal attitudes towards working mothers. We then used binary logistic regression models for the multivariate analyses. Statistical significance was assumed at the *p* > 0.05 level. Effect modification was assessed by stratifying the sample and using interaction terms in Stata. An observed change in odds ratios of ≥ 10% was taken to indicate effect modification. Analyses were preceded by the “svy” function in Stata version 15.1. Results of the logistic regressions are reported in odds ratios which refer to the ratio of the odds of the outcome event in the exposed group compared to the odds in the unexposed group.

## Results

A total of 13,115 children whose mothers were the main respondent participated in the first three sweeps. Data on unintentional injury and ethnicity were available for 13,021 participants. Complete data were available for 12,717 participants on all covariates used in the multivariate analyses. It was deemed appropriate to proceed using complete-case analysis because of the low level of missingness among the variables in the final model [27].

Table 1 displays the characteristics of the full analytic sample, and the sample stratified by ethnic group. Characteristics including reference categories can be viewed in supplementary material S1. Tables showing associations between ethnic group and covariates are available in the supplementary material (Tables S5 – S7). Living in social housing was most prevalent among Black African and Black Caribbean families. Apart from children from Indian backgrounds, minority ethnic children were more likely to experience poverty and live in poor quality housing.

**Table 1:** Characteristics of the sample by ethnic group

		Total sample		White		Indian		Pakistani		Bangladeshi		Black Caribbean		Black African		Other ethnic group	
		*N*	%	*N*	%	*N*	%	*N*	%	*N*	%	*N*	%	*N*	%	*N*	%
**Sociodemographic characteristics**																	
**Household income**	Above 60% median	8657	*68.1*	7919	*72.6*	199	*67.0*	102	*21.5*	33	*17.5*	63	*47.0*	77	*37.2*	264	*52.0*
**Maternal education**	High	4312	*33.9*	3812	*35.0*	105	*35.4*	58	*12.2*	18	*9.5*	50	*37.3*	75	*36.4*	194	*38.0*
**Parents working**	At least one	10,566	*83.1*	9272	*85.0*	267	*89.9*	347	*7301*	134	*70.9*	85	*63.4*	105	*50.7*	356	*69.7*
**Mother born in UK**	Yes	11,218	*88.2*	10,423	*95.6*	127	*42.8*	191	*40.2*	23	*12.2*	103	*76.9*	38	*18.4*	313	*61.3*
**Religion**	None	5472	*4301*	5191	*47.7*	19	*6.4*	11	*2.3*	5	*2.7*	43	*32.1*	10	*4.9*	193	*37.8*
	Christian	5986	*47.1*	5621	*51.6*	4	*1.4*	0	*0.0*	1	*0.5*	86	*64.2*	114	*55.3*	160	*31.3*
	Muslim	885	*7.0*	24	*0.2*	51	*17.1*	454	*95.6*	178	*94.2*	4	*3.0*	80	*38.8*	94	*18.4*
	Hindu and Sikh	286	*2.3*	4	*0.0*	219	*73.7*	7	*1.5*	4	*2.1*	0	*0.00*	0	*0.00*	52	*10.2*
	Other	76	*0.6*	53	*0.5*	4	*1.4*	3	*0.6*	1	*0.5*	1	*1.8*	2	*1.0*	12	*2.4*
**Household composition**	Two parents	10,637	*83.6*	9207	*84.4*	279	*93.9*	431	*90.7*	178	*94.2*	65	*48.5*	118	*57.0*	359	*70.3*
**Housing characteristics**																	
**Housing tenure**	Homeowner	8788	69.1	7741	*71.0*	260	*87.5*	360	*75.8*	93	*49.2*	43	*32.1*	40	*19.3*	251	*49.1*
	Local authority	1969	15.5	1601	*14.7*	13	*4.4*	43	*9.1*	54	*28.6*	42	*31.3*	91	*44.0*	125	*24.5*
	Housing association	1073	8.4	808	*7.4*	14	*4.7*	41	*8.6*	26	*13.8*	43	*32.1*	61	*29.5*	80	*15.7*
	Private rent	887	7.0	754	*6.9*	10	*3.4*	31	*6.5*	16	*8.5*	6	*4.5*	15	*7.3*	55	*10.8*
**Rooms per person**	Less than one	1159	*9.1*	712	*6.5*	43	*14.5*	142	*29.9*	103	*54.5*	22	*16.4*	72	*34.8*	65	*12.7*
	One to two	8881	*69.8*	7707	*70.6*	218	*73.4*	316	*66.5*	82	*43.4*	94	*70.2*	118	*57.0*	352	*68.9*
	Two or more	2677	*21.1*	2491	*22.8*	36	*12.1*	17	*3.6*	4	*2.1*	18	*13.4*	17	*8.2*	94	*18.4*
**Damp**	None or not much	11,849	*93.2*	10,214	*93.7*	278	*93.6*	429	*90.3*	163	*86.2*	117	*87.3*	182	*87.9*	466	*91.2*
**Central heating**	Yes	12,000	*94.4*	10,328	*94.7*	285	*96.0*	404	*8501*	168	*88.9*	131	*97.8*	204	*98.6*	480	*93.9*
**Access to a garden**	Yes, sole use	11,543	*90.8*	10,116	*92.8*	269	*90.6*	428	*90.1*	123	*65.1*	89	*66.4*	116	*56.0*	402	*78.7*
	Yes, shared	318	*2.5*	252	*2.3*	4	*1.4*	16	*3.4*	5	*2.7*	6	*4.5*	11	*5.3*	24	*4.7*
	No	856	*6.7*	536	*4.9*	24	*8.1*	31	*6.5*	61	*32.3*	39	*29.1*	80	*38.7*	85	*16.6*
**Main storey**	Below second floor	12,326	*97.2*	10,678	*98.2*	291	*98.7*	471	*99.6*	143	*75.7*	112	*83.6*	157	*75.9*	474	*93.3*
**Building type**	House/Bungalow	11,508	*90.5*	10,068	*92.3*	281	*94.6*	442	*93.1*	122	*64.6*	85	*63.4*	113	*54.6*	397	*77.7*
	Flat/Maisonette	1168	*9.2*	803	*7.4*	13	*4.4*	32	*6.7*	66	*34.9*	49	*36.6*	94	*45.4*	111	*21.7*
	Room/Bedsit/Other	41	*0.3*	33	*0.3*	3	*1.0*	1	*0.2*	1	*0.5*	0	*0.0*	0	*0.0*	3	*0.6*
**Perception of area safety**	Most safe	11,058	*87.0*	9626	*88.3*	237	*79.8*	390	*82.1*	155	*82.0*	98	*73.1*	160	*77.3*	392	*76.7*
**Play area safety**	Safe	11,621	*98.4*	10,024	*98.5*	266	*97.4*	418	*98.6*	153	*96.8*	121	*98.4*	180	*97.3*	459	*98.3*
**Overall housing quality**	High quality	8832	*69.5*	8017	*73.5*	176	*59.3*	211	*44.4*	40	*21.2*	53	*39.6*	63	*30.4*	272	*53.2*
**Parental and supervision variables**																	
**Maternal age at child’s birth**	11–19	924	*7.3*	823	*7.6*	6	*2.0*	24	*5.1*	13	*6.9*	7	*5.2*	10	*4.8*	41	*8.0*
	20–29	5653	*44.5*	4716	*43.3*	163	*54.9*	316	*66.5*	136	*72.0*	49	*36.6*	65	*61.4*	208	*40.7*
	30–39	5845	*46.0*	5119	*47.0*	125	*42.1*	127	*26.7*	39	*20.6*	69	*51.5*	117	*56.5*	249	*48.7*
	40+	295	*2.3*	246	*2.3*	3	*1.0*	8	*1.7*	1	*0.5*	9	*6.7*	15	*7.3*	13	*2.5*
**Grandparental co-residence**	No	12,190	*95.9*	10,608	*97.3*	222	*74.8*	386	*81.3*	159	*84.1*	130	*97.0*	199	*96.1*	486	*95.1*
**Any other adults in home**	No	12,313	*96.8*	10,670	*97.9*	263	*88.6*	412	*86.7*	163	*86.3*	129	*96.3*	193	*93.2*	483	*95.5*
**Number of siblings**	None	3169	*24.9*	2806	*25.7*	74	*24.9*	55	*11.6*	28	*14.8*	28	*20.9*	37	*17.9*	141	*27.6*
	One	5791	*45.5*	5122	*47.0*	129	*43.4*	164	*34.5*	49	*25.9*	56	*42.8*	57	*27.5*	214	*41.9*
	Two or more	3757	*29.5*	2976	*27.3*	94	*31.7*	256	*53.9*	112	*59.3*	50	*37.3*	113	*54.6*	156	*30.5*
**Household chaos**	Least chaotic	6108	*48.0*	5065	*46.5*	195	*65.7*	297	*61.9*	101	*53.4*	74	*55.2*	121	*58.5*	258	*50.5*
**Any smoking in home**	No	7622	*59.9*	6341	*58.2*	240	*80.8*	340	*71.6*	144	*76.2*	79	*59.0*	184	*88.9*	294	*57.5*
**Maternal alcohol use**	Never/Rarely	7325	*57.6*	5747	*52.7*	271	*91.3*	469	*98.7*	188	*99.5*	93	*69.4*	196	*94.7*	361	*70.7*
**Maternal time with friends**	Regular contact	10,252	*80.6*	8954	*82.1*	191	*64.3*	320	*67.4*	143	*75.7*	93	*69.4*	157	*75.9*	394	*77.1*
**Maternal time with parent(s)**	Regularly see parent(s)	9758	*76.8*	8860	*81.3*	126	*42.4*	249	*52.5*	100	*53.2*	86	*64.2*	50	*24.2*	287	*56.2*
**Good area to bring up children**	Good	11,507	*90.7*	10,000	*91.9*	262	*88.8*	395	*84.2*	158	*85.0*	101	*76.5*	165	*79.7*	426	*83.9*
**Satisfaction with local area**	Satisfied	11,527	*90.6*	9980	*91.5*	264	*88.9*	409	*86.1*	167	*88.4*	107	*79.9*	171	*82.6*	429	*84.0*
**Maternal social capital**	High	11,079	*87.4*	9787	*90.0*	205	*69.5*	338	*72.2*	146	*78.9*	90	*68.2*	127	*61.4*	386	*76.0*
**Values to instill and parenting practices**																	
**Values to instil independence**	Yes	12,240	*99.4*	10,768	*99.6*	226	*97.8*	375	*97.7*	129	*91.5*	130	*100.0*	155	*96.9*	457	*99.4*
**Values: obedience and respect**	Yes	12,103	*99.2*	10,613	*99.2*	227	*99.1*	390	*99.5*	143	*99.3*	126	*100*	165	*99.4*	439	*98.4*
**Values: art of negotiation**	Yes	11,569	*97.4*	10,196	*97.5*	209	*96.3*	350	*96.7*	126	*94.0*	117	*96.7*	151	*96.8*	420	*96.1*
**Values: Respect for elders**	Yes	12,276	*99.6*	10,753	*99.6*	235	*100*	393	*100*	146	*100*	130	*99.2*	166	*100*	453	*99.6*
**Values: Doing well at school**	Yes	12,220	*99.4*	10,694	*99.3*	232	*99.2*	393	*100*	145	*100*	131	*100*	167	*100*	458	*99.8*
**Values: Religious values**	Yes	6583	*59.5*	5303	*55.1*	213	*94.7*	383	*97.7*	134	*94.4*	102	*85.0*	157	*94.6*	291	*70.5*
**Attitude to working mothers**	Favourable/indifferent	9065	*78.3*	8334	*80.1*	129	*67.5*	121	*44.2*	38	*48.1*	93	*77.5*	75	*60.5*	275	*70.0*
**Maternal parenting style**	Structured	5251	*42.8*	4583	*42.6*	101	*42.8*	144	*36.8*	61	*41.5*	75	*57.25*	79	*47.9*	208	*45.3*
**Any screen time**	None	150	*1.2*	112	*1.0*	2	*0.7*	17	*3.6*	8	*4.2*	2	*1.5*	2	*1.0*	7	*1.4*
**Regular bedtime**	Always/sometimes	11,748	*92.4*	10,104	*92.7*	278	*93.6*	430	*90.5*	174	*92.1*	118	*88.1*	182	*87.9*	462	*90.4*

Reference categories of binary variables were included, Full table annexed in supplementary material S2

All minority ethnic groups were more likely to report the mother being born outside of the UK compared to White families and the strongest associations were evident in Pakistani, Bangladeshi, and Black African households. South Asian mothers were more likely to be Muslim, Hindu, and Sikh, while Black Caribbean and Black African mothers were more likely to be Christian and Muslim. South Asian mothers were younger at the birth of the cohort member, while Black Caribbean and Black African mothers were older. South Asian families were the most likely to co-reside with a grandparent and to have another non-parent adult living in the home. Minority ethnic families reported less chaotic homes, less smoking, and less alcohol use than their White counterparts. Minority ethnicity mothers, especially Pakistani, Bangladeshi, and Black African mothers, were more likely than White mothers to agree that instilling religious values in their children was important. These two groups were also more likely to agree with the statement that ‘a child is likely to suffer if his or her mother works before he/she starts school.’

When stratified by the mother’s birthplace (see supplementary materials S2 – S4) the trends evident in Table 1 were reinforced. Minority ethnicity mothers born abroad were more likely to report an income below the 60% median. First generation Black African mothers were less likely to live in a household with any working parent (45%) compared to UK-born mothers (80%). UK-born mothers regardless of ethnicity were less likely to be religious. Apart from Caribbean families, all first-generation mothers were more likely to be members of two parent families. First generation South Asian mothers were more likely to live in multigenerational households. Alcohol use was less common for mothers born-abroad and religious values were also more important to born abroad mothers. Apart from Caribbean mothers, agreement with the idea that a mother returning to work before the child attends school is harmful was higher among born-abroad mothers.

Table 2 displays the proportions of injury by ethnic group and the univariate associations between ethnic group and injury. Pakistani and Bangladeshi, Black African, and those in the ‘other’ ethnicity group were less likely than White children to sustain an injury requiring medical attention.

**Table 2:** Proportion of children injured by ethnic group and unadjusted association between injury and ethnic group (6 category ethnicity variable)

	Full sample	No injury		Any injury		OR	95% CI
	N	N	%	N	%		
Total	12,717	9,269	72.9	3,448	27.1		
White	10,904	7,854	72.0	3,050	28.0	1.00	-
Indian	297	230	77.4	67	22.6	0.73	0.53–1.01
Pakistani and Bangladeshi	664	514	77.4	150	22.6	0.80*	0.66–0.98
Black Caribbean	134	101	75.4	33	24.6	0.97	0.65–1.46
Black African	207	174	84.0	33	15.9	0.46**	0.29–0.74
Other	511	396	77.5	115	22.5	0.65**	0.51–0.82

** *p* < 0.01, *p < 0.05, *n* = 12,717

Univariate analysis (Table 3) showed that social housing and private renting were associated with an increase in injury compared to owner-occupied housing. Poverty, low maternal education, no parent working, lone parenthood, and low housing quality were all associated with injury. Having a mother born outside the UK was associated with a lower likelihood of injury in children compared to those whose mother was born within the UK (OR = 0.71, 95% CI = 0.62, 0.83). Muslim children were less likely than White children to be injured (OR = 0.70, 95% CI = 0.58, 0.85).

Younger maternal age, living in a chaotic home, a household with a smoker, and living in a maternal-rated poor area to bring up children were associated with injury. Having more adults in the home was associated with a lower, but not statistically significant likelihood of injury. Having low contact with parents and endorsing religious values were associated with marginally lower odds of injury.

**Table 3:** Univariate associations between injury and covariates

	Any injury	
	OR	95% CI
Local authority	1.24**	(1.09–1.40)
Housing association	1.22*	(1.03–1.44)
Private rent	1.25*	(1.04–1.50)
Poverty	1.19**	(1.08–1.31)
Low maternal education	1.16**	(1.06–1.27)
No parent working	1.22**	(1.09–1.38)
Mother born outside UK	0.71**	(0.62–0.83)
Christian	0.99	(0.90–1.09)
Muslim	0.70**	(0.58–0.85)
Hindu and Sikh	0.76	(0.54–1.06)
Other	1.12	(0.66–1.89)
Single parent home	1.25**	(1.09–1.43)
Low housing quality	1.14**	(1.04–1.26)
Maternal age 11–19	1.27**	(1.08–1.50)
20–29	1.23**	(1.12–1.35)
40+	0.97	(0.71–1.32)
Grandparental co-residence	0.85	(0.66–1.10)
Another adult resident	0.84	(0.64–1.12)
High chaos	1.27**	(1.15–1.41)
Smoking in home	1.12*	(1.02–1.23)
Maternal frequent alcohol use	0.99	(0.91–1.09)
One sibling	1.01	(0.92–1.11)
Two or more siblings	1.05	(0.92–1.18)
Maternal no time spent with friends	0.91	(0.81–1.01)
Maternal no or low contact with parents	0.90*	(0.81–0.99)
Poor area to bring up children	1.17*	(1.03–1.34)
Low satisfaction with local area	1.09	(0.94–1.27)
Low social capital	1.06	(0.94–1.19)
Values to instil – independence	0.68	(0.39–1.17)
Obedience and respect	1.16	(0.70–1.92)
Art of negotiation	0.97	(0.73–1.29)
Respect for elders	0.83	(0.42–1.63)
Doing well at school	1.27	(0.72–2.25)
Religious values	0.91*	(0.83–1.00)
Maternal negative attitude to working mothers	1.05	(0.93–1.19)
Unstructured	1.02	(0.93–1.12)
Any screentime	1.18	(0.78–1.78)
Irregular bedtime	1.13	(0.96–1.34)

** *p* < 0.01, * *p* < 0.05

When stratified by mother’s birthplace, there was evidence of effect modification (S8): Pakistani or Bangladeshi children whose mother was born in the UK were no less likely to be injured than White children whose mother was born in the UK. However, Pakistani or Bangladeshi children whose mother was born outside the UK were much less likely to be injured. The disparity in the odds ratios (ORs) in the stratified analysis was large.

When stratified by mother’s birthplace, there was a large difference between the ORs for Black Caribbean mothers although the confidence intervals were wide.

Table 4 shows the multivariate association between injury, socioeconomic factors, ethnicity and maternal migrant status, and parental and household level factors. In the first model, male sex (OR = 1.34, 95% CI = 1.22–1.46) and living in local authority (OR = 1.18, 95% CI = 1.03–1.35) and private rented housing (OR = 1.22, 95% CI = 1.01–1.47) were associated with injury. A Wald test confirmed the addition of ethnic group, migrant status of the mother, and their interaction contributed to the model fit *F*(5, 385) = 2.32, *p* = 0.040. Compared to White children whose mothers were born in the UK, Pakistani or Bangladeshi children whose mother was born outside the UK were less likely to be injured (OR = 0.57, 95% CI = 0.37–0.86). Children in the ‘other’ ethnic group were less likely to be injured that their White counterparts and the odds were even lower for those whose mothers were born outside the UK (OR = 0.54, 95% CI = 0.30–0.97). The main effect of ethnic group on injury was not significant for most groups, but the odds ratios for Black African children were notably low. Further adjusting for maternal age, household chaos, smoking in the home, and household composition contributed further to the model *F*(6, 384) = 5.19, *p* < 0.001. Of these variables, only household chaos was associated with injury (OR = 1.22, 95% CI = 1.10–1.36). There was also a notable difference between Black Caribbean children whose mothers were born in the UK and those who were born abroad, although the ORs were not significant.

**Table 4:** Adjusted odds for unintentional childhood injuries adjusted for sex socioeconomic factors, ethnicity and migrant status, their interaction, and parent and household factors

	Adjusted for sex and socioeconomic factors		Ethnicity and mother’s migrant status		Adjusted for parental and household factors	
	OR	95% CI	OR	95% CI	OR	95% CI
**Sex**						
Female	1.00	-	1.00	-	1.00	-
Male	1.34**	1.22–1.46	1.34**	1.22–1.47	1.33**	1.21–1.46
**OECD Equivalised Income**						
Above 60% median income	1.00	-	1.00	-	1.00	-
Below 60% median income	1.09	0.97–1.21	1.14*	1.00-1.28	1.07	0.95–1.21
**Housing tenure**						
Owner-occupied	1.00	-	1.00	-	1.00	-
Local authority	1.18*	1.03–1.35	1.20*	1.04–1.38	1.13	0.97–1.32
Housing association	1.18	0.98–1.41	1.20	1.00-1.44	1.12	0.93–1.36
Private rent	1.22*	1.01–1.47	1.21*	1.00-1.46	1.15	0.95–1.40
**Ethnic group**						
White			1.00	-	1.00	-
Indian			0.71	0.45–1.14	0.72	0.45–1.16
Pakistani or Bangladeshi			1.10	0.84–1.43	1.13	0.86–1.48
Black Caribbean			1.08	0.70–1.68	1.10	0.72–1.69
Black African			0.35	0.12–1.02	0.37	0.13–1.09
Other			0.73*	0.55–0.97	0.74*	0.55–0.98
**Mother born in UK**						
Yes			1.00	-	1.00	-
No			0.98	0.77–1.23	1.01	0.80–1.27
**Interaction: Ethnic group x mother born in UK**						
White x mother born in UK			1.00	-	1.00	-
Indian x mother born outside UK			1.12	0.55–2.25	1.12	
Pakistani or Bangladeshi x mother born outside UK			0.57**	0.37–0.86	0.55**	0.45–1.16
Black Caribbean x mother born outside UK			0.23	0.05–1.06	0.23	0.86–1.48
Black African x mother born outside UK			1.22	0.41–3.63	1.18	0.72–1.69
“Other” ethnic group			0.54*	0.30–0.97	0.54*	0.13–1.09
**Maternal age**						
30–39					1.00	-
11–19					1.05	0.88–1.25
20–29					1.17**	1.05–1.30
40+					1.00	0.73–1.38
**Household chaos**						
Least chaotic					1.00	-
Most chaotic					1.22**	1.10–1.36
**Any smoking in the home**						
No					1.00	-
Yes					0.97	0.88–1.08
**Household Composition**						
Two parent household					1.00	-
One parent household					1.13	0.96–1.33

** *p* < 0.01, * *p* < 0.05,n = 12,717

The models including the interaction between ethnic group and migrant status was not statistically superior to the model without interaction terms (Table S9), however, it is evident that migrant status does alter the relationship between ethnic group and injury and does so differently for different groups. For example, the relationship between Pakistani and Bangladeshi ethnicity and lower injury odds is only evident for children whose mothers were born outside the UK, while for Black African children, those whose mothers were born in the UK had lower odds of injury.

## Discussion

Much of the research on social inequalities in childhood injury is based on area of residence for the comparison of rates among social groups, consistently finding that injury rates are higher among children in relatively deprived areas compared to affluent areas [28]. While questions arise on the relative distribution of risk of those emanating from residence in a poor household or living in a poor neighbourhood [29]. Nevertheless, while reviews of epidemiological studies of unintentional injury indicate the presence of a social gradient, there is considerable variation in the level of risk, the causes of injury, and their contexts. Thus, while a gradient may exist it is commonly influenced by multiple factors [30]. Importantly, the theory of “fundamental causes” also predicts that international and/or local variations in the relationship between socioeconomic factors and injury may be explained by compensatory policies – welfare and housing improvement programmes, and/or educational schemes on health and safety [5].

In univariate analyses, Pakistani and Bangladeshi children (OR = 0.81, 95% CI = 0.66, 0.98) and Black African children (OR = 0.46, 95% CI = 0.28, 0.75) were significantly less likely to sustain an unintentional injury than White children. Children in the ‘other’ ethnicity group also showed reduced injury risk (OR = 0.75, 95% CI = 0.57, 0.98). Having a mother born outside the UK was independently associated with lower injury risk (OR = 0.71, 95% CI = 0.62, 0.83).

However, when maternal migrant status and its interaction with ethnicity were included in multivariate models, the main effects of ethnicity became non-significant for most groups. The protective effect was primarily evident among children whose mothers were born outside the UK: Pakistani or Bangladeshi children with foreign-born mothers had significantly lower injury odds (OR = 0.57, 95% CI = 0.37–0.86), as did children in the ‘other’ ethnic group with foreign-born mothers (OR = 0.54, 95% CI = 0.30–0.97). Notably, Pakistani or Bangladeshi children whose mothers were born in the UK showed no significant difference in injury risk compared to White children with UK-born mothers, suggesting that migrant status explains the observed protective effect.

Despite these groups experiencing higher rates of poverty and poor-quality housing, the association between ethnicity and reduced injury risk persisted even after controlling for socioeconomic circumstances. Among the household and parenting variables examined, only household chaos was significantly associated with increased injury risk (OR = 1.22, 95% CI = 1.10–1.36) in the final model. While we observed substantial differences between ethnic groups in parenting practices, health behaviours, and value systems, these factors did not statistically explain the ethnic variation in injury.

Our findings that children from some ethnic groups were less likely to sustain an unintentional injury than their White counterparts align with previous research in the UK. Campbell et al. [10] found non-White ethnicity was associated with lower injury among five-year-olds. We expanded on these findings by disaggregating ethnicity and migrant status. Epidemiological studies using administrative healthcare data also suggest lower injury incidence among Black and South Asian children in the UK [11, 12] possibly explained by higher bone density and lower physical activity [6]. However, Grgic et al. [13] controlled for these factors and found that these groups were less likely to sustain fractures in a Dutch population. Our study, while focused on broader unintentional injuries and not just fractures, confirms this finding.

Nevertheless, we noted strong sociocultural differences across ethnic or national groups that may influence risk of childhood injuries, factors that relate to household composition and parenting styles. Thus, multigenerational or multi-adult extended households are more common in some ethnic communities. For example, in the UK, there is consistent evidence that Pakistani, Bangladeshi, and Indian households are much more likely to include three or more generations under one roof compared to White British households. While household composition (among other characteristics) of minority communities tends to converge with the host nation over time, this is not consistent across groups. Thus, Bangladeshi and Pakistani households remain large and multigenerational, relative to others. Moreover, while Caribbean and African women are increasingly economically active, this is not the case for their Pakistani and Bangladeshi counterparts who are then more likely to be at home, different positions that are unlikely to be explained by discrimination. The English Housing Survey noted that British Bangladeshis and Pakistanis are 12 times more likely than those from White British households to experience overcrowding (British Bangladeshi 24% vs. White British 2%) [31]. Whatever the explanation for this variation in size of ethnic households, a potential effect is to increase the number of available adults to supervise children; a ratio that appears to offset the risks associated with economic disadvantage.

Parenting, household composition, health behaviours, and value systems differed between ethnic groups and these differences were more pronounced in mothers who were born abroad. For example, South Asian children were more likely to live in two-parent families than White children and an even greater proportion of South Asian children whose mothers were born outside the UK lived in two-parent families. Similar trends were observed for multigenerational living. While these variables did not statistically explain ethnic variation in the likelihood of injury in this sample, there is evidence from other studies that they could play a role. Black African, Pakistani, and Bangladeshi women in this cohort were more likely to exhibit a constellation of traditional values and behaviours – they were more religious, less likely to consume alcohol or smoke, and more likely to agree with the sentiment that mothers should stay home before their child reaches school-age, and these patterns were more pronounced in born-abroad mothers. Hazardous alcohol consumption by household adults has been associated with a higher risk of poisonings, thermal injuries, and long-bone fractures in children, while maternal alcohol use during pregnancy has been linked to raised surface falls in children aged 0–2 years [32–35].

Renzaho et al. (2011) used focus group discussions with African (Somali, Sudanese, and Ethiopian) families living in Melbourne and found African parents were more willing to control their children’s behaviour through close monitoring, strict boundary-setting, and the threat of physical punishment [36]. This may explain some of the lower injury risk in African families.

Multigenerational households have been previously associated with lower unintentional injury risk and this effect seems to be more pronounced in boys [18, 19]. Two-parent households and married parents have also been associated with lower injury risk in other studies [34, 37]. These factors were higher among minority ethnic women and higher again in mothers born abroad.

Studies of the relationship between ethnicity and unintentional childhood injuries generally fail to control for the migrant status of the children or family. However, as stated by other academics, ethnicity is a problematic multidimensional epidemiological variable, often employed to cover concepts that don’t neatly overlap. Thus, for research purposes the Millennium Cohort Study ethnicity categories are adopted from those in the UK census which define an ethnic group as having subjective dimensions such as shared values, beliefs, customs and traditions, and language; but has more objective dimensions such as nationality, religious identity, skin colour, and country of origin (self and ancestors) [38].

In our study, having a mother born outside the UK appears protective of childhood unintentional injuries. However, the international evidence is limited and contradictory [15, 39, 40]. Behaviours and lifestyle factors associated with reduced risk of injury in Black African and South Asian households were more pronounced in born-abroad families. For example, South Asian and Black African mothers were more likely to be religious, live with extended family, and were less likely to smoke in the home or drink alcohol and these factors were even more common in born-abroad mothers suggesting acculturation may increase risk of problematic behaviours and injury. Generational changes to health-related behaviour has been noted for all ethnic groups in previous research [41] with second-generation migrants more likely to consume alcohol than the first-generation [42] suggesting that increased alcohol consumption among the second-generation may explain ethnic minorities’ generational health deterioration. This is supported by qualitative research with first-generation Pakistani mothers who described themselves as more traditional than second-generation mothers [43]. These mothers provided more proximal and infant-led care, including always keeping their baby with them and obtained more communal support from female relatives than White British mothers.

There may also be unique protective factors in migrant families which were not measured in this study. Migrant families in an environment with which they are not yet familiar, may rely more strongly on family support and be more alert to hazards. Additionally, born-abroad mothers may parent in ways which are more common in their country of origin than their host country. For example, Cooke and Waite (2016) found that while African migrant mothers often adapted their parenting styles to align more closely with British cultural norms, they identified values such as respect and discipline as being more significant in their parenting compared to British culture [44].

Alternatively, the disparity in medically attended injuries among first-generation migrants may be a result of defining medical attention differently or be less likely to seek treatment. Evidence from Ireland shows that healthcare utilisation (including GP visits and emergency department attendances) for children of non-EU immigrants was lower than in children of Irish-born or UK-born parents [45]. This is a finding which appears to be replicated across high income host countries and the disparity in healthcare use has been attributed to factors such as language barriers, a lack of knowledge of the host healthcare system and structural barriers to accessing healthcare such as access to transport or registering with care providers [46]. In our study, respondents were asked if their child had sustained an injury for which they “had been taken to the doctor, health centre, or hospital.” It is reasonable to extrapolate that lower general healthcare utilisation among migrants may account for at least some of relationship between migrant status, ethnicity, and injury in the present study. However, as previously noted, even severe injuries which warrant hospital admission still display these patterns [12] and these injuries are less likely to be impacted by lower healthcare utilisation in general. It is unclear whether lower healthcare utilisation for childhood unintentional injuries is an example of inequity in healthcare provision or is a result of a lower burden of unintentional injuries and this distinction warrants further research.

Strengths: This study used a large, nationally representative dataset which allowed us to disaggregate unintentional injuries by ethnic group and mothers’ migrant status. We were also able to include a wide range of covariates such as environmental, economic, and cultural indicators. Further, the longitudinal aspect of the study design means that exposures were measured before the outcome and strengthening the evidence for the direction of the relationships.

Limitations: First, the format of a birth cohort study may not be the most appropriate format to assess the cultural and behavioural parenting differences – these practices may be too nuanced and subjective to statistically compare. Further, asylum-seeking and refugee children are at higher risk of injury, particularly burns [47, 48]. These groups were excluded from the present study because respondents were identified through the child benefit system, therefore some of the most vulnerable migrant groups are not represented.

Data were collected between 2003 and 2006. While this may limit immediate applicability, the fundamental demographic and cultural patterns observed in ethnic minority communities have remained relatively consistent. However, changes in housing quality and healthcare accessibility over the intervening period may influence the magnitude of associations. Future research with contemporary data would strengthen confidence in these findings.

Unintentional injuries were based on maternal reporting of injuries and may be subject to recall bias. Vulnerable groups unfamiliar with the healthcare system and social welfare may be unwilling to report potentially undesirable information. Social desirability bias may also operate differently across cultural groups. Families from certain ethnic backgrounds, particularly recent migrants, may be more reluctant to report incidents that could be perceived as reflecting poor parental supervision or child safety practices. However, evidence suggests there is good agreement between maternal report and medical records [49] and the alignment of our findings with administrative healthcare studies suggests that genuine protective effects, rather than reporting artifacts alone, contribute to the observed differences [11, 12].

Future research: While this study provides evidence that the mother’s migrant status is a key factor in the lower injury rates among minority ethnic children in the UK, the exact mechanisms remain unclear. Cultural practices and values, particularly those associated with first-generation migrants from South Asia and Africa, likely play a significant role in shaping child safety behaviours. Further research is needed to explore these cultural dimensions in greater depth and to better understand the protective factors contributing to the lower injury rates observed among minority ethnic groups in the UK. The results highlight the importance of considering the role of migration when investigating the relationship between ethnic group and unintentional childhood injury and disaggregating data by ethnic group rather than treating minority ethnicity as a homogenous group.

These results have important implications for policymaking, discussed below, however caution should be exercised when applying these findings to contexts outside the UK. The findings can be generalised to the UK population because the Millennium Cohort Study is a nationally representative data source. There is some evidence that there is similar ethnic and migrant variation in childhood unintentional injury in other European contexts [13], but policymakers should take care when extrapolating these results to all high income, western societies. Patterns of migration vary globally, and social, economic, and structural factors such as socialised healthcare are all likely to impact the relationship between ethnicity, migration, and childhood injury risk.

Implications for policymaking: The National Institute for Health and Care Excellence (NICE) guidance on interventions for unintentional childhood injuries in the home (PH30) includes a recommendation to account for cultural and religious beliefs when carrying out home safety checks and installation of safety equipment [21]. However, cultural competency is not routinely integrated into national policymaking frameworks; for example, neither Public Health England’s document on ‘Reducing unintentional injuries in and around the home among children under five’, nor Northern Ireland’s ‘Home Accident Prevention Strategy’ (2015–2025) mention cultural or religious beliefs in their prevention strategies [22]. This gap is notable given international guidance from the WHO Global Action Plan on promoting the health of refugees and migrants (2019–2030) that emphasises the importance of culturally sensitive approaches to health promotion and injury prevention, specifically calling for “refugee-sensitive and migrant-sensitive health policies” and “culturally appropriate health services” [50].

While UK policy acknowledges that inequality and material deprivation increase the risk of unintentional injuries and recommend interventions targeted at the most at-risk groups, our findings suggest this approach may be insufficient. The lower injury rates we observed in Pakistani, Bangladeshi, and Black African families despite their higher rates of poverty and poor-quality housing indicate that current risk stratification models may miss important protective factors present in these communities. Moreover, our findings demonstrate that ethnicity and maternal migration status are related but distinct factors that require separate policy consideration. Therefore, we recommend that future injury prevention strategies should distinguish between ethnicity-focused and migration-focused interventions while acknowledging their intersection. Further, given that first-generation migrants show stronger protective effects that may diminish over generations, we recommend that injury prevention interventions should aim to understand and integrate protective cultural approaches to child-rearing rather than viewing minority status solely as a risk factor.

## Conclusion

Despite living in poorer quality housing and experiencing greater socioeconomic disadvantage, children from certain minority ethnic backgrounds in the UK are less likely to sustain unintentional injuries than their White counterparts. However, this protective effect is primarily evident among children whose mothers were born outside the UK, suggesting that maternal migrant status, rather than ethnicity per se, is the key protective factor. Pakistani, Bangladeshi, and children in the ‘other’ ethnic group with foreign-born mothers showed significantly reduced injury risk, whilst those with UK-born mothers demonstrated no significant difference compared to White children.

Although we observed substantial cultural and behavioural differences between ethnic groups, particularly pronounced among foreign-born mothers, these factors did not statistically explain the observed variation in injury rates. This suggests that protective mechanisms may operate through unmeasured pathways not captured in our study design.

The findings emphasise the importance of disaggregating ethnicity and migrant status in injury prevention research. Future research should investigate the specific factors that contribute to lower injury rates among first-generation migrant families and examine whether observed differences reflect genuine protective effects or differential healthcare utilisation patterns. Understanding these mechanisms could inform injury prevention strategies that harness protective cultural factors rather than viewing minority status solely through a deficit lens.

## Electronic supplementary material

Supplementary Material 1

## Data Availability

Millennium Cohort Study data are available to download from the UK Data Service: https://ukdataservice.ac.uk/.

## Abbreviations

UK: United Kingdom
MCS: Millennium Cohort Study
NHS: National Health Service
OR: Odds ratio

## References

[1] Godson R. Reducing unintentional injuries in and around the home among children under five years. Community Pract. 2014;87(8):12–3. 25226699

[2] Gallagher L, Breslin G, Leavey G, Curran E, Rosato M. Determinants of unintentional injuries in preschool age children in high-income countries: a systematic review. Child Care Health Dev. 2024;50(1):e13161. 37555597 10.1111/cch.13161

[3] Mayes S, Roberts MC, Stough CO. Risk for household safety hazards: socioeconomic and sociodemographic factors. J Saf Res. 2014;51:87–92. 10.1016/j.jsr.2014.09.002 25453181

[4] Laflamme L. Social inequality in injury risks: knowledge accumulated and plans for the future. National Institute of Public Health. 1998.

[5] Willson AE. Fundamental causes’ of health disparities: a comparative analysis of Canada and the united States. Int Sociol. 2009;24(1):93–113.

[6] Towner E, Dowswell T, Errington G, Burkes M, Towner J. Injuries in children aged 0–14 years and inequalities. London: Health Development Agency; 2005.

[7] Bourdieu P. The forms of capital. In: Richardson J, editor. Handbook of theory and research for the sociology of education. New York: Greenwood; 1986. pp. 241–58.

[8] Gulliver K. Racial discrimination in UK housing has a long history and deep roots [Internet]. British Politics and Policy at LSE. 2017 [cited 2023 Jun 13]. Available from: https://blogs.lse.ac.uk/politicsandpolicy/racial-discrimination-in-housing/

[9] Rogaly K, Elliott J, Baxter D. What’s causing structural racism in housing? Joseph Rowntree Foundation; 2021.

[10] Campbell M, Lai ET, Pearce A, Orton E, Kendrick D, Wickham S, et al. Understanding pathways to social inequalities in childhood unintentional injuries: findings from the UK millennium cohort study. BMC Pediatr. 2019;19:1–17. 31088415 10.1186/s12887-019-1514-7 PMC6518796

[11] Moon RJ, Harvey NC, Curtis EM, de Vries F, van Staa T, Cooper C. Ethnic and geographic variations in the epidemiology of childhood fractures in the United Kingdom. Bone. 2016;85:9–14. 26802259 10.1016/j.bone.2016.01.015 PMC4841386

[12] Tobin MD, Milligan J, Shukla R, Crump B, Burton PR. South Asian ethnicity and risk of childhood accidents: an ecological study at enumeration district level in Leicester. J Public Health. 2002;24(4):313–8. 10.1093/pubmed/24.4.313 12546210

[13] Grgic O, Chung K, Shevroja E, Trajanoska K, Uitterlinden AG, Wolvius EB, et al. Fractures in school age children in relation to sex and ethnic background: the generation R study. Bone. 2019;121:227–31. 30677542 10.1016/j.bone.2019.01.019

[14] Rechel B, Mladovsky P, Ingleby D, Mackenbach JP, McKee M. Migration and health in an increasingly diverse Europe. Lancet. 2013;381(9873):1235–45. 23541058 10.1016/S0140-6736(12)62086-8

[15] Laursen B, Møller H. Unintentional injuries in children of Danish and foreign-born mothers. Scand J Public Health. 2009;37(6):577–83. 19470692 10.1177/1403494809105793

[16] Prevoo MJ, Tamis-LeMonda CS. Parenting and globalization in Western countries: explaining differences in parent–child interactions. Curr Opin Psychol. 2017;15:33–9. 28813265 10.1016/j.copsyc.2017.02.003

[17] Morioka H, Itani O, Jike M, Nakagome S, Otsuka Y, Ohida T. Risk factors at birth predictive of subsequent injury among Japanese preschool children: a nationwide 5-year cohort study. J Dev Behav Pediatr. 2018;39(5):424. 29557859 10.1097/DBP.0000000000000558

[18] Tanskanen AO, Danielsbacka M. Association between grandparental co-residence and early childhood injury in the UK. Child Indic Res. 2017;10(3):825–37.

[19] Schnitzer PG, Dowd MD, Kruse RL, Morrongiello BA. Supervision and risk of unintentional injury in young children. Inj Prev. 2015;21(e1):e63-70. 24848998 10.1136/injuryprev-2013-041128 PMC4293371

[20] Tan KT, Prowse PM, Falder S. Ethnic differences in burn mechanism and severity in a UK paediatric population. Burns. 2012;38(4):551–5. 22079622 10.1016/j.burns.2011.10.005

[21] National Institute for Health and Care Excellence. Unintentional injuries in the home: interventions for under 15s: NICE public health guidance 30. [Internet]. 2010. Available from: guidance.nice.org.uk/PH30

[22] Public Health England. Reducing unintentional injuries in and around the home among children under five years. London; 2018.

[23] Hansen K. Millennium cohort study: a guide to the datasets. London: UCL Institute of Education; 2014.

[24] Shepherd P, Gilbert E. Millennium cohort study: Ethical review and consent. Centre for Longitudinal studies. 2019.

[25] Jayaweera H, Quigley MA. Health status, health behaviour and healthcare use among migrants in the UK: evidence from mothers in the millennium cohort study. Soc Sci Med. 2010;71(5):1002–10. 20624665 10.1016/j.socscimed.2010.05.039

[26] Hosmer DW Jr, Lemeshow S, Sturdivant RX. Applied logistic regression. Wiley; 2013.

[27] Langkamp DL, Lehman A, Lemeshow S. Techniques for handling missing data in secondary analyses of large surveys. Acad Pediatr. 2010;10(3):205–10. 20338836 10.1016/j.acap.2010.01.005 PMC2866831

[28] Faelker T, Pickett W, Brison RJ. Socioeconomic differences in childhood injury: a population based epidemiologic study in Ontario, Canada. Inj Prev. 2000;6(3):203–8. 11003186 10.1136/ip.6.3.203 PMC1730634

[29] Seah R, Lystad RP, Curtis K, Mitchell R. Socioeconomic variation in injury hospitalisations in Australian children ≤ 16 years: a 10-year population-based cohort study. BMC Public Health. 2018;18(1):1336. 10.1186/s12889-018-6242-7 PMC6278126 30509222

[30] Laflamme L, Hasselberg M, Burrows S. 20 years of research on socioeconomic inequality and Children′s—Unintentional injuries Understanding the Cause-Specific evidence at hand. Int J Pediatr. 2010;2010(1):819687. 20706660 10.1155/2010/819687 PMC2913857

[31] Department for Levelling Up. Housing, & Communities. English Housing Survey 2021 to 2022: headline report. 2022.

[32] Baker R, Orton E, Tata LJ, Kendrick D. Risk factors for long-bone fractures in children up to 5 years of age: a nested case–control study. Arch Dis Child. 2015;100(5):432–7. 25398446 10.1136/archdischild-2013-305715 PMC4413839

[33] Culasso M, Porta D, Brescianini S, Gagliardi L, Michelozzi P, Pizzi C, et al. Unintentional injuries and potential determinants of falls in young children: results from the Piccolipiu Italian birth cohort. PLoS ONE. 2022;17(10):e0275521. 36191030 10.1371/journal.pone.0275521 PMC9529104

[34] Orton E, Kendrick D, West J, Tata LJ. Independent risk factors for injury in pre-school children: three population-based nested case-control studies using routine primary care data. PLoS One. 2012;7(4): e35193. 22496906 10.1371/journal.pone.0035193 PMC3320631

[35] Auger N, Chadi N, Low N, Ayoub A, Lo E, Luu TM. Maternal substance use disorders and accidental drug poisonings in children. Am J Prev Med. 2022;62(3):360–6. 34802817 10.1016/j.amepre.2021.09.007

[36] Renzaho AMN, Green J, Mellor D, Swinburn B. Parenting, family functioning and lifestyle in a new culture: the case of African migrants in melbourne, victoria, Australia. Child Family Social Work. 2011;16(2):228–40.

[37] Panico L, Bartley M, Kelly YJ, McMunn A, Sacker A. Family structure trajectories and early child health in the UK: pathways to health. Soc Sci Med. 2019;232:220–9. 31102932 10.1016/j.socscimed.2019.05.006

[38] Karlsen S, Nazroo JY. Agency and structure: the impact of ethnic identity and racism on the health of ethnic minority people. Sociol Health Illn. 2002;24(1):1–20.

[39] Karimi N, Beiki O, Mohammadi R. Risk of fatal unintentional injuries in children by migration status: a nationwide cohort study with 46 years’ follow-up. Inj Prev. 2015;21(e1):e80–7. 24108384 10.1136/injuryprev-2013-040883

[40] Saunders NR, Macpherson A, Guan J, Guttmann A. Unintentional injuries among refugee and immigrant children and youth in Ontario, Canada: a population-based cross-sectional study. Inj Prev. 2018;24(5):337–43. 28951486 10.1136/injuryprev-2016-042276

[41] Wang S, Li S. Exploring generational differences of British ethnic minorities in smoking behavior, frequency of alcohol consumption, and dietary style. Int J Environ Res Public Health. 2019;16(12):2241. 31242661 10.3390/ijerph16122241 PMC6616626

[42] Allen JD, Caspi C, Yang M, Leyva B, Stoddard AM, Tamers S, et al. Pathways between acculturation and health behaviors among residents of low-income housing: the mediating role of social and contextual factors. Soc Sci Med. 2014;123:26–36. 25462602 10.1016/j.socscimed.2014.10.034 PMC4425350

[43] Crane D. BradICS: bradford infant care study: a qualitative study of infant care practices and unexpected infant death in an urban multi-cultural UK population [PhD Thesis]. Durham University; 2014.

[44] Cook J, Waite L. I think i’m more free with them’—Conflict, negotiation and change in intergenerational relations in African families living in Britain. J Ethnic Migration Stud. 2016;42(8):1388–402.

[45] Mohan G. The influence of caregiver’s migration status on child’s use of healthcare services: evidence from Ireland. Sociol Health Illn. 2021;43(3):557–74. 33636049 10.1111/1467-9566.13239

[46] Thandi I, Salami B, Ladha T. Factors affecting emergency department use among pediatric immigrants and refugees: A qualitative narrative review. Int Health Trends Perspect. 2023;3(2):156–69.

[47] Dempsey M, Orr D. Are paediatric burns more common in asylum seekers? An analysis of paediatric burn admissions. Burns. 2006;32(2):242–25. 16448770 10.1016/j.burns.2005.09.004

[48] Friedl NK, Muensterer OJ. Special aspects in pediatric surgical inpatient care of refugee children: a comparative cohort study. Children. 2019;6(5): 62. 31052220 10.3390/children6050062 PMC6560456

[49] Cummings P, Rivara FP, Thompson R, Reid RJ. Ability of parents to recall the injuries of their young children. Inj Prev. 2005;11(1):43–7. 15691989 10.1136/ip.2004.006833 PMC1730187

[50] World Health Organization. WHO global action plan on promoting the health of refugees and migrants 2019–2030. Geneva; 2024.

