# Supplementary material for: Socioeconomic circumstances, ethnicity, migration and unintentional early childhood injuries: an analysis of the UK millennium cohort study
Source: Inj Epidemiol. 2025 Sep 2;12:54. doi: 10.1186/s40621-025-00603-y (PMC12403302; doi:10.1186/s40621-025-00603-y)
Supplement: Supplementary file 1 — Supplementary Material 1 [file 40621_2025_603_MOESM1_ESM.docx]

| Table S1. Characteristics of the sample by ethnic group full table | | | | | | | | | | | | | | | | |
| --- | --- | --- | --- | --- | --- | --- | --- | --- | --- | --- | --- | --- | --- | --- | --- | --- |
|  | **Total sample** | | **White** | | **Indian** | | **Pakistani** | | **Bangladeshi** | | **Black**  **Caribbean** | | **Black**  **African** | | **Other**  **ethnic group** | |
|  | **N** | **%** | **N** | **%** | **N** | **%** | **N** | **%** | **N** | **%** | **N** | **%** | **N** | **%** | **N** | **%** |
| Sociodemographic |  |  |  |  |  |  |  |  |  |  |  |  |  |  |  |  |
| **Household income** |  |  |  |  |  |  |  |  |  |  |  |  |  |  |  |  |
| Above 60% median | 8657 | *68.1* | 7919 | *72.6* | 199 | *67.0* | 102 | *21.5* | 33 | *17.5* | 63 | *47.0* | 77 | *37.2* | 264 | *52.0* |
| Below 60% median | 4060 | *31.9* | 2985 | *27.4* | 98 | *33.0* | 373 | *78.5* | 156 | *82.5* | 71 | *53.0* | 130 | *62.8* | 247 | *48.3* |
| **Maternal education** |  |  |  |  |  |  |  |  |  |  |  |  |  |  |  |  |
| Level 4 & 5 | 4312 | *33.9* | 3812 | *35.0* | 105 | *35.4* | 58 | *12.2* | 18 | *9.5* | 50 | *37.3* | 75 | *36.4* | 194 | *38.0* |
| Level 3 and below | 8401 | *66.1* | 7089 | *65.0* | 192 | *64.7* | 417 | *87.8* | 171 | *90.5* | 84 | *62.7* | 131 | *63.6* | 317 | *62.0* |
| **Parents working** |  |  |  |  |  |  |  |  |  |  |  |  |  |  |  |  |
| At least one | 10566 | *83.1* | 9272 | *85.0* | 267 | *89.9* | 347 | *7301* | 134 | *70.9* | 85 | *63.4* | 105 | *50.7* | 356 | *69.7* |
| None | 2151 | *16.9* | 1632 | *15.0* | 30 | *10.1* | 128 | *27.0* | 55 | *29.1* | 49 | *36.6* | 102 | *49.3* | 155 | *30.3* |
| **Mother born in UK** |  |  |  |  |  |  |  |  |  |  |  |  |  |  |  |  |
| Yes | 11218 | *88.2* | 10423 | *95.6* | 127 | *42.8* | 191 | *40.2* | 23 | *12.2* | 103 | *76.9* | 38 | *18.4* | 313 | *61.3* |
| No | 1499 | *11.8* | 481 | *4.4* | 170 | *57.2* | 284 | *59.8* | 166 | *87.8* | 31 | *23.1* | 169 | *81.6* | 198 | *38.8* |
| **Religion** |  |  |  |  |  |  |  |  |  |  |  |  |  |  |  |  |
| None | 5472 | *4301* | 5191 | *47.7* | 19 | *6.4* | 11 | *2.3* | 5 | *2.7* | 43 | *32.1* | 10 | *4.9* | 193 | *37.8* |
| Christian | 5986 | *47.1* | 5621 | *51.6* | 4 | *1.4* | 0 | *0.0* | 1 | *0.5* | 86 | *64.2* | 114 | *55.3* | 160 | *31.3* |
| Muslim | 885 | *7.0* | 24 | *0.2* | 51 | *17.1* | 454 | *95.6* | 178 | *94.2* | 4 | *3.0* | 80 | *38.8* | 94 | *18.4* |
| Hindu and Sikh | 286 | *2.3* | 4 | *0.0* | 219 | *73.7* | 7 | *1.5* | 4 | *2.1* | 0 | *0.00* | 0 | *0.00* | 52 | *10.2* |
| Other | 76 | *0.6* | 53 | *0.5* | 4 | *1.4* | 3 | *0.6* | 1 | *0.5* | 1 | *1.8* | 2 | *1.0* | 12 | *2.4* |
| **Household composition** |  |  |  |  |  |  |  |  |  |  |  |  |  |  |  |  |
| Two parents | 10637 | *83.6* | 9207 | *84.4* | 279 | *93.9* | 431 | *90.7* | 178 | *94.2* | 65 | *48.5* | 118 | *57.0* | 359 | *70.3* |
| One parent | 2080 | *1634* | 1697 | *15.6* | 18 | *6.1* | 44 | *9.3* | 11 | *5.8* | 69 | *51.5* | 89 | *43.0* | 152 | *29.8* |
| **Housing characteristics** |  |  |  |  |  |  |  |  |  |  |  |  |  |  |  |  |
| **Housing tenure** |  |  |  |  |  |  |  |  |  |  |  |  |  |  |  |  |
| Homeowner | 8788 | 69.1 | 7741 | *71.0* | 260 | *87.5* | 360 | *75.8* | 93 | *49.2* | 43 | *32.1* | 40 | *19.3* | 251 | *49.1* |
| Local authority | 1969 | 15.5 | 1601 | *14.7* | 13 | *4.4* | 43 | *9.1* | 54 | *28.6* | 42 | *31.3* | 91 | *44.0* | 125 | *24.5* |
| Housing association | 1073 | 8.4 | 808 | *7.4* | 14 | *4.7* | 41 | *8.6* | 26 | *13.8* | 43 | *32.1* | 61 | *29.5* | 80 | *15.7* |
| Private rent | 887 | 7.0 | 754 | *6.9* | 10 | *3.4* | 31 | *6.5* | 16 | *8.5* | 6 | *4.5* | 15 | *7.3* | 55 | *10.8* |
| **Rooms per person** |  |  |  |  |  |  |  |  |  |  |  |  |  |  |  |  |
| Less than one | 1159 | *9.1* | 712 | *6.5* | 43 | *14.5* | 142 | *29.9* | 103 | *54.5* | 22 | *16.4* | 72 | *34.8* | 65 | *12.7* |
| One to two | 8881 | *69.8* | 7707 | *70.6* | 218 | *73.4* | 316 | *66.5* | 82 | *43.4* | 94 | *70.2* | 118 | *57.0* | 352 | *68.9* |
| Two or more | 2677 | *21.1* | 2491 | *22.8* | 36 | *12.1* | 17 | *3.6* | 4 | *2.1* | 18 | *13.4* | 17 | *8.2* | 94 | *18.4* |
| **Damp** |  |  |  |  |  |  |  |  |  |  |  |  |  |  |  |  |
| None or not much | 11849 | *93.2* | 10214 | *93.7* | 278 | *93.6* | 429 | *90.3* | 163 | *86.2* | 117 | *87.3* | 182 | *87.9* | 466 | *91.2* |
| Some or a great problem | 868 | *6.8* | 690 | *6.3* | 19 | *6.4* | 46 | *9.7* | 26 | *13.8* | 17 | *12.7* | 25 | *12.1* | 45 | *8.8* |
| **Central heating** |  |  |  |  |  |  |  |  |  |  |  |  |  |  |  |  |
| Yes | 12000 | *94.4* | 10328 | *94.7* | 285 | *96.0* | 404 | *8501* | 168 | *88.9* | 131 | *97.8* | 204 | *98.6* | 480 | *93.9* |
| No | 717 | *5.6* | 579 | *5.3* | 12 | *4.0* | 71 | *15.0* | 21 | *11.1* | 3 | *2.2* | 3 | *1.5* | 31 | *6.1* |
| **Access to a garden** |  |  |  |  |  |  |  |  |  |  |  |  |  |  |  |  |
| Yes, sole use | 11543 | *90.8* | 10116 | *92.8* | 269 | *90.6* | 428 | *90.1* | 123 | *65.1* | 89 | *66.4* | 116 | *56.0* | 402 | *78.7* |
| Yes, shared | 318 | *2.5* | 252 | *2.3* | 4 | *1.4* | 16 | *3.4* | 5 | *2.7* | 6 | *4.5* | 11 | *5.3* | 24 | *4.7* |
| No | 856 | *6.7* | 536 | *4.9* | 24 | *8.1* | 31 | *6.5* | 61 | *32.3* | 39 | *29.1* | 80 | *38.7* | 85 | *16.6* |
| **Main storey** |  |  |  |  |  |  |  |  |  |  |  |  |  |  |  |  |
| Below second floor | 12326 | *97.2* | 10678 | *98.2* | 291 | *98.7* | 471 | *99.6* | 143 | *75.7* | 112 | *83.6* | 157 | *75.9* | 474 | *93.3* |
| Second floor and above | 355 | *2.8* | 197 | *1.8* | 4 | *1.4* | 2 | *0.4* | 46 | *24.3* | 22 | *16.4* | 50 | *24.2* | 34 | *6.7* |
| **Building type** |  |  |  |  |  |  |  |  |  |  |  |  |  |  |  |  |
| House/Bungalow | 11508 | *90.5* | 10068 | *92.3* | 281 | *94.6* | 442 | *93.1* | 122 | *64.6* | 85 | *63.4* | 113 | *54.6* | 397 | *77.7* |
| Flat/Maisonette | 1168 | *9.2* | 803 | *7.4* | 13 | *4.4* | 32 | *6.7* | 66 | *34.9* | 49 | *36.6* | 94 | *45.4* | 111 | *21.7* |
| Room/Bedsit/Other | 41 | *0.3* | 33 | *0.3* | 3 | *1.0* | 1 | *0.2* | 1 | *0.5* | 0 | *0.0* | 0 | *0.0* | 3 | *0.6* |
| **Perception of area safety** |  |  |  |  |  |  |  |  |  |  |  |  |  |  |  |  |
| Most safe | 11058 | *87.0* | 9626 | *88.3* | 237 | *79.8* | 390 | *82.1* | 155 | *82.0* | 98 | *73.1* | 160 | *77.3* | 392 | *76.7* |
| Least safe | 1659 | *13.1* | 1278 | *11.7* | 60 | *20.2* | 85 | *17.9* | 34 | *18.0* | 36 | *26.9* | 47 | *22.7* | 119 | *23.3* |
| **Play area safety** |  |  |  |  |  |  |  |  |  |  |  |  |  |  |  |  |
| Safe | 11621 | *98.4* | 10024 | *98.5* | 266 | *97.4* | 418 | *98.6* | 153 | *96.8* | 121 | *98.4* | 180 | *97.3* | 459 | *98.3* |
| Unsafe | 187 | *1.6* | 154 | *1.5* | 7 | *2.6* | 6 | *1.4* | 5 | *3.1* | 2 | *1.6* | 5 | *2.7* | 8 | *1.7* |
| **Overall housing quality** |  |  |  |  |  |  |  |  |  |  |  |  |  |  |  |  |
| High quality | 8832 | *69.5* | 8017 | *73.5* | 176 | *59.3* | 211 | *44.4* | 40 | *21.2* | 53 | *39.6* | 63 | *30.4* | 272 | *53.2* |
| Low quality | 3885 | *30.6* | 2887 | *26.5* | 121 | *40.7* | 264 | *55.6* | 149 | *78.8* | 81 | *60.5* | 144 | *69.6* | 239 | *46.8* |
| **Parental and supervision variables** |  |  |  |  |  |  |  |  |  |  |  |  |  |  |  |  |
| **Maternal age at child’s birth** |  |  |  |  |  |  |  |  |  |  |  |  |  |  |  |  |
| 11-19 | 924 | *7.3* | 823 | *7.6* | 6 | *2.0* | 24 | *5.1* | 13 | *6.9* | 7 | *5.2* | 10 | *4.8* | 41 | *8.0* |
| 20-29 | 5653 | *44.5* | 4716 | *43.3* | 163 | *54.9* | 316 | *66.5* | 136 | *72.0* | 49 | *36.6* | 65 | *61.4* | 208 | *40.7* |
| 30-39 | 5845 | *46.0* | 5119 | *47.0* | 125 | *42.1* | 127 | *26.7* | 39 | *20.6* | 69 | *51.5* | 117 | *56.5* | 249 | *48.7* |
| 40+ | 295 | *2.3* | 246 | *2.3* | 3 | *1.0* | 8 | *1.7* | 1 | *0.5* | 9 | *6.7* | 15 | *7.3* | 13 | *2.5* |
| **Grandparental co-residence** |  |  |  |  |  |  |  |  |  |  |  |  |  |  |  |  |
| No | 12190 | *95.9* | 10608 | *97.3* | 222 | *74.8* | 386 | *81.3* | 159 | *84.1* | 130 | *97.0* | 199 | *96.1* | 486 | *95.1* |
| Yes | 527 | *4.1* | 296 | *2.7* | 75 | *25.3* | 89 | *18.7* | 30 | *15.9* | 4 | *3.0* | 8 | *3.9* | 25 | *4.9* |
| **Any other adults in home** |  |  |  |  |  |  |  |  |  |  |  |  |  |  |  |  |
| No | 12313 | *96.8* | 10670 | *97.9* | 263 | *88.6* | 412 | *86.7* | 163 | *86.3* | 129 | *96.3* | 193 | *93.2* | 483 | *95.5* |
| Yes | 404 | *3.2* | 234 | *2.2* | 34 | *11.5* | 63 | *13.3* | 26 | *13.8* | 5 | *3.7* | 14 | *6.7* | 28 | *5.5* |
| **Number of siblings** |  |  |  |  |  |  |  |  |  |  |  |  |  |  |  |  |
| None | 3169 | *24.9* | 2806 | *25.7* | 74 | *24.9* | 55 | *11.6* | 28 | *14.8* | 28 | *20.9* | 37 | *17.9* | 141 | *27.6* |
| One | 5791 | *45.5* | 5122 | *47.0* | 129 | *43.4* | 164 | *34.5* | 49 | *25.9* | 56 | *42.8* | 57 | *27.5* | 214 | *41.9* |
| Two or more | 3757 | *29.5* | 2976 | *27.3* | 94 | *31.7* | 256 | *53.9* | 112 | *59.3* | 50 | *37.3* | 113 | *54.6* | 156 | *30.5* |
| **Household chaos** |  |  |  |  |  |  |  |  |  |  |  |  |  |  |  |  |
| Least chaotic | 6108 | *48.0* | 5065 | *46.5* | 195 | *65.7* | 297 | *61.9* | 101 | *53.4* | 74 | *55.2* | 121 | *58.5* | 258 | *50.5* |
| Most chaotic | 6609 | *52.0* | 5839 | *53.6* | 102 | *34.3* | 181 | *38.1* | 88 | *46.6* | 60 | *44.8* | 86 | *41.6* | 253 | *49.5* |
| **Any smoking in home** |  |  |  |  |  |  |  |  |  |  |  |  |  |  |  |  |
| No | 7622 | *59.9* | 6341 | *58.2* | 240 | *80.8* | 340 | *71.6* | 144 | *76.2* | 79 | *59.0* | 184 | *88.9* | 294 | *57.5* |
| Yes | 5095 | *40.1* | 4563 | *41.9* | 57 | *19.2* | 135 | *28.4* | 45 | *23.8* | 55 | *41.0* | 23 | *11.1* | 217 | *42.5* |
| **Maternal alcohol use** |  |  |  |  |  |  |  |  |  |  |  |  |  |  |  |  |
| Never/Rarely | 7325 | *57.6* | 5747 | *52.7* | 271 | *91.3* | 469 | *98.7* | 188 | *99.5* | 93 | *69.4* | 196 | *94.7* | 361 | *70.7* |
| Often | 5392 | *42.4* | 5157 | *47.3* | 26 | *7.8* | 6 | *1.3* | 1 | *0.5* | 41 | *30.6* | 11 | *5.3* | 150 | *29.4* |
| **Maternal time with friends** |  |  |  |  |  |  |  |  |  |  |  |  |  |  |  |  |
| No friends or no time with friends | 2465 | *19.4* | 1950 | *17.9* | 106 | *35.7* | 155 | *32.6* | 46 | *24.3* | 41 | *30.6* | 50 | *24.2* | 117 | *22.9* |
| Regular contact | 10252 | *80.6* | 8954 | *82.1* | 191 | *64.3* | 320 | *67.4* | 143 | *75.7* | 93 | *69.4* | 157 | *75.9* | 394 | *77.1* |
| **Maternal time with parent(s)** |  |  |  |  |  |  |  |  |  |  |  |  |  |  |  |  |
| Never or rarely | 2953 | *23.2* | 2040 | *18.7* | 171 | *57.6* | 225 | *47.5* | 88 | *46.8* | 48 | *35.8* | 157 | *75.9* | 224 | *43.8* |
| Regularly see parent(s) | 9758 | *76.8* | 8860 | *81.3* | 126 | *42.4* | 249 | *52.5* | 100 | *53.2* | 86 | *64.2* | 50 | *24.2* | 287 | *56.2* |
| **Good area to bring up children** |  |  |  |  |  |  |  |  |  |  |  |  |  |  |  |  |
| Poor | 1175 | *9.3* | 885 | *8.1* | 33 | *11.2* | 74 | *15.8* | 28 | *15.1* | 31 | *23.5* | 42 | *20.3* | 82 | *16.1* |
| Good | 11507 | *90.7* | 10000 | *91.9* | 262 | *88.8* | 395 | *84.2* | 158 | *85.0* | 101 | *76.5* | 165 | *79.7* | 426 | *83.9* |
| **Satisfaction with local area** |  |  |  |  |  |  |  |  |  |  |  |  |  |  |  |  |
| Not satisfied | 1190 | *9.4* | 924 | *8.5* | 33 | *11.1* | 66 | *13.9* | 22 | *11.6* | 27 | *20.2* | 36 | *17.4* | 82 | *16.1* |
| Satisfied | 11527 | *90.6* | 9980 | *91.5* | 264 | *88.9* | 409 | *86.1* | 167 | *88.4* | 107 | *79.9* | 171 | *82.6* | 429 | *84.0* |
| **Maternal social capital** |  |  |  |  |  |  |  |  |  |  |  |  |  |  |  |  |
| Low | 1597 | *12.6* | 1094 | *10.1* | 90 | *30.1* | 130 | *27.8* | 39 | *21.1* | 42 | *31.8* | 80 | *38.7* | 122 | *24.0* |
| High | 11079 | *87.4* | 9787 | *90.0* | 205 | *69.5* | 338 | *72.2* | 146 | *78.9* | 90 | *68.2* | 127 | *61.4* | 386 | *76.0* |
| **Values to instill and parenting practices** |  |  |  |  |  |  |  |  |  |  |  |  |  |  |  |  |
| **Values to instil independence** |  |  |  |  |  |  |  |  |  |  |  |  |  |  |  |  |
| Yes | 12240 | *99.4* | 10768 | *99.6* | 226 | *97.8* | 375 | *97.7* | 129 | *91.5* | 130 | *100.0* | 155 | *96.9* | 457 | *99.4* |
| No | 73 | *0.6* | 39 | *0.4* | 5 | *2.2* | 9 | *2.3* | 12 | *8.5* | 0 | *0.0* | 5 | *3.1* | 3 | *0.7* |
| **Values: obedience and respect** |  |  |  |  |  |  |  |  |  |  |  |  |  |  |  |  |
| Yes | 12103 | *99.2* | 10613 | *99.2* | 227 | *99.1* | 390 | *99.5* | 143 | *99.3* | 126 | *100* | 165 | *99.4* | 439 | *98.4* |
| No | 100 | *0.8* | 87 | *0.8* | 2 | *0.9* | 2 | *0.5* | 1 | *0.7* | 0 | *0.0* | 1 | *0.6* | 7 | *1.6* |
| **Values: art of negotiation** |  |  |  |  |  |  |  |  |  |  |  |  |  |  |  |  |
| Yes | 11569 | *97.4* | 10196 | *97.5* | 209 | *96.3* | 350 | *96.7* | 126 | *94.0* | 117 | *96.7* | 151 | *96.8* | 420 | *96.1* |
| No | 311 | *2.6* | 257 | *2.5* | 8 | *3.7* | 12 | *3.3* | 8 | *6.0* | 4 | *3.3* | 5 | *3.2* | 17 | *3.9* |
| **Values: Respect for elders** |  |  |  |  |  |  |  |  |  |  |  |  |  |  |  |  |
| Yes | 12276 | *99.6* | 10753 | *99.6* | 235 | *100* | 393 | *100* | 146 | *100* | 130 | *99.2* | 166 | *100* | 453 | *99.6* |
| No | 43 | *0.4* | 0 | *0.0* | 0 | *0.0* | 0 | *0.0* | 0 | *0.0* | 1 | *0.8* | 0 | *0.0* | 2 | *0.4* |
| **Values: Doing well at school** |  |  |  |  |  |  |  |  |  |  |  |  |  |  |  |  |
| Yes | 12220 | *99.4* | 10694 | *99.3* | 232 | *99.2* | 393 | *100* | 145 | *100* | 131 | *100* | 167 | *100* | 458 | *99.8* |
| No | 71 | *0.7* | 2 | *0.9* | 0 | *0.0* | 0 | *0.0* | 0 | *0.0* | 0 | *0.0* | 0 | *0.0* | 1 | *0.2* |
| **Values: Religious values** |  |  |  |  |  |  |  |  |  |  |  |  |  |  |  |  |
| Yes | 6583 | *59.5* | 5303 | *55.1* | 213 | *94.7* | 383 | *97.7* | 134 | *94.4* | 102 | *85.0* | 157 | *94.6* | 291 | *70.5* |
| No | 4491 | *40.6* | 4313 | *44.9* | 12 | *5.3* | 9 | *2.3* | 8 | *5.6* | 18 | *15.0* | 9 | *5.4* | 122 | *29.5* |
| **Attitude to working mothers** |  |  |  |  |  |  |  |  |  |  |  |  |  |  |  |  |
| Favourable/indifferent | 9065 | *78.3* | 8334 | *80.1* | 129 | *67.5* | 121 | *44.2* | 38 | *48.1* | 93 | *77.5* | 75 | *60.5* | 275 | *70.0* |
| Unfavourable | 2518 | *21.7* | 2068 | *19.9* | 62 | *32.5* | 153 | *55.8* | 41 | *51.9* | 27 | *22.5* | 49 | *39.5* | 118 | *30.0* |
| **Maternal parenting style** |  |  |  |  |  |  |  |  |  |  |  |  |  |  |  |  |
| Structured | 5251 | *42.8* | 4583 | *42.6* | 101 | *42.8* | 144 | *36.8* | 61 | *41.5* | 75 | *57.25* | 79 | *47.9* | 208 | *45.3* |
| Unstructured | 7033 | *57.2* | 6172 | *57.4* | 135 | *57.2* | 247 | *63.2* | 86 | *58.5* | 56 | *42.8* | 86 | *52.1* | 251 | *54.7* |
| **Any screen time** |  |  |  |  |  |  |  |  |  |  |  |  |  |  |  |  |
| None | 150 | *1.2* | 112 | *1.0* | 2 | *0.7* | 17 | *3.6* | 8 | *4.2* | 2 | *1.5* | 2 | *1.0* | 7 | *1.4* |
| Any screentime | 12566 | *98.8* | 10791 | *99.0* | 295 | *99.3* | 458 | *96.4* | 181 | *95.8* | 132 | *98.5* | 205 | *99.0* | 504 | *98.6* |
| **Regular bedtime** | 12716 |  |  |  |  |  |  |  |  |  |  |  |  |  |  |  |
| Always/sometimes | 11748 | *92.4* | 10104 | *92.7* | 278 | *93.6* | 430 | *90.5* | 174 | *92.1* | 118 | *88.1* | 182 | *87.9* | 462 | *90.4* |
| Never | 968 | *7.6* | 799 | *7.3* | 19 | *6.4* | 45 | *9.5* | 15 | *7.9* | 16 | *11.9* | 25 | *12.1* | 49 | *9.6* |

| Table S2. Socioeconomic variables by ethnic group and birthplace of mother | | | | | | | | | | | | | | |
| --- | --- | --- | --- | --- | --- | --- | --- | --- | --- | --- | --- | --- | --- | --- |
|  | Full Sample | | White | | Indian | | Pakistani and Bangladeshi | | Black Caribbean | | Black African | | Other | |
|  | UK-born | Born-abroad | UK-born | Born-abroad | UK-born | Born-abroad | UK-born | Born-abroad | UK-  born | Born-abroad | UK-born | Born-abroad | UK-born | Born-abroad |
|  | N  *%* | N  *%* | N  % | N  *%* | N  *%* | N  % | N  *%* | N  *%* | N  % | N  *%* | N  *%* | N  % | N  *%* | N  *%* |
| **Maternal age at child’s birth** |  |  |  |  |  |  |  |  |  |  |  |  |  |  |
| 11-19 | 899 | 53 | 830 | 16 | 4 | 3 | 24 | 16 | 3 | 4 | 2 | 8 | 36 | 6 |
|  | *7.8* | *3.4* | *7.8* | *3.2* | *3.1* | *1.7* | *10.5* | *3.3* | *2.9* | *12.9* | *5.0* | *4.5* | *11.2* | *2.9* |
| 20-29 | 5080 | 741 | 4645 | 176 | 87 | 85 | 154 | 335 | 45 | 5 | 16 | 56 | 133 | 84 |
|  | *44.3* | *47.1* | *43.7* | *35.3* | *66.4* | *47.5* | *67.5* | *69.5* | *42.9* | *16.1* | *40.0* | *31.6* | *41.3* | *40.8* |
| 30-39 | 5232 | 727 | 4923 | 291 | 40 | 88 | 50 | 122 | 53 | 17 | 21 | 98 | 145 | 111 |
|  | *45.7* | *46.2* | *46.3* | *58.4* | *30.5* | *49.2* | *21.9* | *25.3* | *50.5* | *54.8* | *52.5* | *55.4* | *45.0* | *53.9* |
| 40+ | 251 | 52 | 238 | 15 | 0 | 3 | 0 | 9 | 4 | 5 | 1 | 15 | 8 | 5 |
|  | *2.2* | *3.3* | *2.2* | *3.0* | *0.0* | *1.7* | *0.0* | *1.9* | *3.8* | *16.1* | *2.5* | *8.5* | *2.5* | *2.4* |
| **Household income** |  |  |  |  |  |  |  |  |  |  |  |  |  |  |
| Above 60% median | 8040 | 786 | 7654 | 397 | 93 | 115 | 50 | 96 | 47 | 18 | 24 | 57 | 172 | 103 |
|  | *70.2* | *49.9* | *72.0* | *79.7* | *71.0* | *64.3* | *21.9* | *19.9* | *44.8* | *58.1* | *60.0* | *32.2* | *53.3* | *50.0* |
| Below 60% median | 3413 | 788 | 2972 | 101 | 38 | 64 | 178 | 387 | 58 | 13 | 16 | 120 | 151 | 103 |
|  | *29.8* | *50.1* | *28.0* | *20.3* | *29.0* | *35.8* | *78.1* | *80.1* | *55.2* | *41.9* | *40.0* | *67.8* | *46.8* | *50.0* |
| **Maternal education** |  |  |  |  |  |  |  |  |  |  |  |  |  |  |
| Level 4 & 5 | 3914 | 494 | 3644 | 247 | 50 | 58 | 33 | 49 | 42 | 9 | 27 | 50 | 118 | 81 |
|  | *34.2* | *31.4* | *34.3* | *49.6* | *38.2* | *32.4* | *14.5* | *10.1* | *40.0* | *29.0* | *67.5* | *28.4* | *36.7* | *39.3* |
| Level 3 and below | 7540 | 1080 | 6985 | 251 | 81 | 121 | 194 | 435 | 63 | 22 | 13 | 126 | 204 | 125 |
|  | *65.8* | *68.6* | *65.7* | *50.4* | *61.8* | *67.6* | *85.5* | *89.9* | *60.0* | *71.0* | *32.5* | *71.6* | *63.4* | *60.7* |
| **Parents working** |  |  |  |  |  |  |  |  |  |  |  |  |  |  |
| At least one | 9617 | 1199 | 9010 | 446 | 119 | 159 | 168 | 349 | 65 | 22 | 32 | 79 | 223 | 144 |
|  | *83.9* | *76.1* | *84.7* | *89.6* | *90.8* | *88.8* | *73.7* | *72.1* | *61.9* | *71.0* | *80.0* | *44.6* | *69.0* | *69.9* |
| None | 1846 | 376 | 1626 | 52 | 12 | 20 | 60 | 135 | 40 | 9 | 8 | 98 | 100 | 62 |
|  | *16.1* | *23.9* | *15.3* | *10.4* | *9.2* | *11.2* | *26.3* | *27.9* | *38.1* | *29.0* | *20.0* | *55.4* | *31.0* | *30.1* |

| *Table S3. Cultural and household variables by ethnic group and birthplace of mother* | | | | | | | | | | | | | | |
| --- | --- | --- | --- | --- | --- | --- | --- | --- | --- | --- | --- | --- | --- | --- |
|  | Full sample | | White | | Indian | | Pakistani and Bangladeshi | | Black Caribbean | | Black African | | Other | |
|  | UK-born | Born-abroad | UK-born | Born-abroad | UK-born | Born-abroad | UK-born | Born-abroad | UK-  born | Born-abroad | UK-born | Born-abroad | UK-born | Born-abroad |
| N  *%* | N  *%* | N  % | N  *%* | N  *%* | N  % | N  *%* | N  *%* | N  % | N  *%* | N  *%* | N  % | N  *%* | N  *%* | N  *%* |
| **Religion** |  |  |  |  |  |  |  |  |  |  |  |  |  |  |
| None | 5333 | 247 | 5106 | 186 | 10 | 9 | 12 | 5 | 36 | 7 | 6 | 6 | 163 | 34 |
|  | *46.6* | *15.7* | *48.1* | *37.4* | *7.6* | *5.0* | *5.3* | *1.0* | *34.6* | *22.6* | *15.0* | *3.4* | *50.6* | *16.5* |
| Christian | 5680 | 441 | 5459 | 286 | 2 | 2 | 1 | 0 | 66 | 21 | 33 | 86 | 119 | 46 |
|  | *49.6* | *28.0* | *51.4* | *57.4* | *1.5* | *1.1* | *0.5* | *0.0* | *63.5* | *67.7* | *82.5* | *48.9* | *37.0* | *22.3* |
| Muslim | 264 | 675 | 5 | 19 | 21 | 32 | 209 | 468 | 1 | 3 | 1 | 82 | 27 | 71 |
|  | *2.3* | *42.9* | *0.05* | *3.8* | *16.0* | *17.9* | *92.1* | *96.7* | *1.0* | *9.7* | *2.5* | *46.6* | *8.4* | *34.5* |
| Hindu and Sikh | 114 | 187 | 2 | 2 | 97 | 133 | 5 | 7 | 0 | 0 | 0 | 0 | 10 | 45 |
|  | *1.0* | *11.9* | *0.02* | *0.4* | *74.1* | *74.3* | *2.2* | *1.5* | *0.0* | *0.0* | *0.0* | *0.0* | *3.1* | *21.8* |
| Other | 55 | 24 | 50 | 5 | 1 | 3 | 0 | 4 | 1 | 0 | 0 | 2 | 3 | 10 |
|  | *0.5* | *1.5* | *0.5* | *1.0* | *0.8* | *1.7* | *0.0* | *0.8* | *1.0* | *0.0* | *0.0* | *1.1* | *1.0* | *4.9* |
| **Household composition** |  |  |  |  |  |  |  |  |  |  |  |  |  |  |
| Two parents | 9518 | 1365 | 8927 | 453 | 118 | 172 | 196 | 455 | 52 | 14 | 23 | 102 | 202 | 169 |
|  | *83.2* | *86.8* | *84.1* | *91.0* | *90.1* | *96.1* | *86.0* | *94.2* | *50.0* | *45.2* | *57.5* | *58.0* | *62.9* | *82.0* |
| One parent | 1924 | 208 | 1691 | 45 | 13 | 7 | 32 | 28 | 52 | 17 | 17 | 74 | 119 | 37 |
|  | *16.8* | *13.2* | *15.9* | *9.0* | *9.9* | *3.9* | *14.0* | *5.8* | *50.0* | *54.8* | *42.5* | *42.1* | *37.1* | *18.0* |
| **Grandparental co-residence** |  |  |  |  |  |  |  |  |  |  |  |  |  |  |
| No | 11097 | 1388 | 10340 | 489 | 104 | 126 | 201 | 380 | 101 | 31 | 39 | 170 | 312 | 192 |
|  | *96.8* | *88.1* | *97.2* | *98.2* | *79.4* | *70.4* | *88.2* | *78.5* | *96.2* | *100.0* | *97.5* | *96.1* | *96.6* | *93.2* |
| Yes | 366 | 187 | 296 | 9 | 27 | 53 | 27 | 104 | 4 | 0 | 1 | 7 | 11 | 14 |
|  | *3.2* | *11.9* | *2.8* | *1.8* | *20.6* | *29.6* | *11.8* | *21.5* | *3.8* | *0.0* | *2.5* | *4.0* | *3.4* | *6.8* |
| **Any other adults in home** |  |  |  |  |  |  |  |  |  |  |  |  |  |  |
| No | 11147 | 1411 | 10382 | 487 | 115 | 152 | 201 | 397 | 101 | 30 | 37 | 161 | 311 | 184 |
|  | *97.5* | *91.1* | *97.8* | *98.2* | *89.8* | *86.4* | *90.1* | *84.1* | *96.2* | *96.8* | *92.54* | *93.6* | *96.6* | *91.1* |
| Yes | 291 | 138 | 238 | 9 | 13 | 24 | 22 | 75 | 4 | 1 | 3 | 11 | 11 | 18 |
|  | *2.5* | *8.9* | *2.2* | *1.8* | *10.2* | *13.6* | *9.9* | *15.9* | *3.8* | *3.2* | *7.5* | *6.4* | *3.4* | *8.9* |

| *Table S4. Supervision and parenting variables by ethnic group and birthplace of mother* | | | | | | | | | | | | | | |
| --- | --- | --- | --- | --- | --- | --- | --- | --- | --- | --- | --- | --- | --- | --- |
|  | Full sample | | White | | Indian | | Pakistani and Bangladeshi | | Black Caribbean | | Black African | | Other | |
|  | UK-born | Born-abroad | UK-born | Born-abroad | UK-born | Born-abroad | UK-born | Born-abroad | UK-  born | Born-abroad | UK-born | Born-abroad | UK-born | Born-abroad |
|  | N  *%* | N  % | N  *%* | N  *%* | N  % | N  *%* | N  *%* | N  % | N  *%* | N  *%* | N  % | N  *%* | N  *%* | N  *%* |
| **Number of siblings** |  |  |  |  |  |  |  |  |  |  |  |  |  |  |
| None | 2949 | 296 | 2731 | 131 | 36 | 42 | 42 | 50 | 21 | 7 | 11 | 28 | 108 | 38 |
|  | *25.7* | *18.8* | *25.7* | *26.3* | *27.5* | *23.5* | *18.4* | *10.3* | *20.0* | *22.6* | *27.5* | *15.8* | *33.4* | *18.5* |
| One | 5331 | 604 | 4992 | 237 | 57 | 75 | 89 | 143 | 50 | 8 | 14 | 48 | 129 | 93 |
|  | *46.5* | *38.4* | *46.9* | *47.6* | *43.5* | *41.9* | *39.0* | *29.6* | *47.6* | *25.8* | *35.0* | *27.1* | *39.9* | *45.2* |
| Two or more | 3183 | 675 | 2913 | 130 | 38 | 62 | 97 | 291 | 34 | 16 | 15 | 101 | 86 | 75 |
|  | *27.8* | *42.9* | *27.4* | *26.1* | *29.0* | *34.6* | *42.5* | *60.1* | *32.4* | *51.6* | *37.5* | *57.1* | *26.6* | *36.4* |
| **Household chaos** |  |  |  |  |  |  |  |  |  |  |  |  |  |  |
| Least chaotic | 5354 | 905 | 4886 | 274 | 89 | 116 | 138 | 285 | 59 | 17 | 31 | 94 | 151 | 119 |
|  | *49.7* | *57.5* | *45.9* | *55.0* | *67.9* | *64.8* | *60.5* | *58.9* | *56.2* | *54.8* | *77.5* | *53.1* | *46.8* | *57.8* |
| Most chaotic | 61.8 | 670 | 5749 | 224 | 42 | 63 | 90 | 199 | 46 | 14 | 9 | 83 | 172 | 87 |
|  | *53.3* | *42.5* | *54.1* | *45.0* | *32.1* | *35.2* | *39.5* | *41.1* | *43.8* | *45.2* | *22.5* | *46.9* | *53.3* | *42.2* |
| **Any smoking in home** |  |  |  |  |  |  |  |  |  |  |  |  |  |  |
| No | 6654 | 1156 | 6143 | 315 | 102 | 148 | 162 | 360 | 59 | 22 | 34 | 159 | 154 | 152 |
|  | *58.1* | *73.4* | *57.8* | *63.3* | *77.9* | *82.7* | *71.1* | *74.4* | *56.2* | *71.0* | *85.0* | *89.8* | *47.7* | *73.8* |
| Yes | 4809 | 419 | 4493 | 183 | 29 | 31 | 66 | 124 | 46 | 9 | 6 | 18 | 169 | 54 |
|  | *42.0* | *26.6* | *42.2* | *36.8* | *22.1* | *17.3* | *29.0* | *25.6* | *43.8* | *29.0* | *15.0* | *10.2* | *52.3* | *26.2* |
| **Maternal alcohol use** |  |  |  |  |  |  |  |  |  |  |  |  |  |  |
| Never/Rarely | 6227 | 1304 | 5585 | 285 | 112 | 171 | 222 | 481 | 70 | 24 | 37 | 169 | 201 | 174 |
|  | *54.3* | *82.8* | *52.5* | *57.2* | *85.5* | *95.5* | *97.4* | *99.4* | *66.7* | *77.4* | *92.5* | *95.5* | *62.2* | *84.5* |
| Often | 5236 | 271 | 5051 | 213 | 19 | 8 | 6 | 3 | 35 | 7 | 3 | 8 | 122 | 32 |
|  | *45.7* | *17.2* | *47.5* | *42.8* | *14.5* | *4.5* | *2.6* | *0.6* | *33.3* | *22.6* | *7.5* | *4.5* | *37.8* | *15.5* |
| **Values: Religious values** |  |  |  |  |  |  |  |  |  |  |  |  |  |  |
| Yes | 5725 | 1033 | 5147 | 270 | 96 | 125 | 188 | 365 | 80 | 24 | 34 | 131 | 180 | 118 |
|  | *56.6* | *84.1* | *54.7* | *64.3* | *90.6* | *98.4* | *94.0* | *98.1* | *84.2* | *88.9* | *91.9* | *95.6* | *64.8* | *81.4* |
| No | 4394 | 195 | 4256 | 150 | 10 | 2 | 12 | 7 | 15 | 3 | 3 | 6 | 98 | 27 |
|  | *43.4* | *15.9* | *45.3* | *35.7* | *9.4* | *1.6* | *6.0* | *1.9* | *15.8* | *11.1* | *8.1* | *4.4* | *35.3* | *18.6* |
| **Attitude to working mothers** |  |  |  |  |  |  |  |  |  |  |  |  |  |  |
| Favourable/indifferent | 8653 | 593 | 8168 | 327 | 79 | 54 | 83 | 82 | 77 | 18 | 27 | 53 | 219 | 59 |
|  | *79.4* | *63.0* | *80.2* | *74.8* | *72.5* | *60.7* | *46.1* | *42.7* | *77.8* | *78.3* | *73.0* | *57.0* | *74.5* | *55.1* |
| Unfavourable | 2247 | 348 | 2013 | 110 | 30 | 35 | 97 | 110 | 22 | 5 | 10 | 40 | 75 | 48 |
|  | *20.6* | *37.0* | *19.8* | *25.2* | *27.5* | *39.3* | *53.9* | *57.3* | *22.2* | *21.7* | *27.0* | *43.0* | *25.5* | *44.9* |
| **Maternal parenting style** |  |  |  |  |  |  |  |  |  |  |  |  |  |  |
| Structured | 4870 | 515 | 4485 | 203 | 53 | 54 | 92 | 126 | 64 | 13 | 24 | 59 | 152 | 60 |
|  | *43.2* | *39.8* | *42.7* | *43.6* | *46.5* | *41.5* | *46.0* | *33.5* | *61.5* | *44.8* | *63.2* | *43.7* | *48.6* | *38.0* |
| Unstructured | 6413 | 779 | 6029 | 263 | 61 | 76 | 108 | 250 | 40 | 16 | 14 | 76 | 161 | 98 |
|  | *56.8* | *60.2* | *57.3* | *56.4* | *53.5* | *58.5* | *54.0* | *66.5* | *38.5* | *55.2* | *36.8* | *56.3* | *51.4* | *62.0* |
| **Any screen time** |  |  |  |  |  |  |  |  |  |  |  |  |  |  |
| None | 123 | 36 | 108 | 9 | 0 | 3 | 9 | 19 | 1 | 1 | 0 | 2 | 5 | 2 |
|  | *1.1* | *2.3* | *1.0* | *1.8* | *0.0* | *1.7* | *4.0* | *3.9* | *1.0* | *3.2* | *0.0* | *1.1* | *1.5* | *1.0* |
| Any screentime | 11339 | 1539 | 10527 | 489 | 131 | 176 | 219 | 465 | 104 | 30 | 40 | 175 | 318 | 204 |
|  | *98.9* | *97.7* | *99.0* | *98.2* | *100.0* | *98.3* | *96.0* | *96.1* | *99.0* | *96.8* | *100.0* | *98.9* | *98.5* | *99.0* |
| **Regular bedtime** |  |  |  |  |  |  |  |  |  |  |  |  |  |  |
| Always/sometimes | 10600 | 1446 | 9842 | 476 | 126 | 165 | 210 | 440 | 92 | 28 | 38 | 153 | 292 | 184 |
|  | *92.5* | *91.8* | *92.5* | *95.6* | *96.2* | *92.2* | *92.1* | *90.9* | *87.6* | *90.3* | *95.0* | *86.4* | *90.4* | *89.3* |
| Never | 862 | 129 | 793 | 22 | 5 | 14 | 18 | 44 | 13 | 3 | 2 | 24 | 31 | 22 |
|  | *7.5* | *8.2* | *7.5* | *4.4* | *3.8* | *7.8* | *7.9* | *9.1* | *12.4* | *9.7* | *5.0* | *13.6* | *9.6* | *10.7* |

| Table S5. Univariate associations between ethnic group and socioeconomic, cultural, and housing variables | | | | | | | | | | |
| --- | --- | --- | --- | --- | --- | --- | --- | --- | --- | --- |
|  | **Indian** | | **Pakistani or**  **Bangladeshi** | | **Black**  **Caribbean** | | **Black**  **African** | | **Other ethnic**  **group** | |
|  | **OR** | **95% CI** | **OR** | **95% CI** | **OR** | **95% CI** | **OR** | **95% CI** | **OR** | **95% CI** |
| Local authority | 0.22** | (0.10 - 0.47) | 0.99 | (0.58 - 1.71) | 3.43** | (1.68 - 7.01) | 8.54** | (4.60 - 15.86) | 2.40** | (1.77 - 3.24) |
| Housing association | 0.33** | (0.15 - 0.73) | 1.25 | (0.83 - 1.88) | 9.01** | (4.72 - 17.18) | 8.53** | (4.37 - 16.65) | 3.03** | (2.23 - 4.12) |
| Private rent | 0.27** | (0.14 - 0.51) | 0.78 | (0.44 - 1.36) | 1.53 | (0.47 - 4.95) | 2.59* | (1.21 - 5.52) | 2.31** | (1.57 - 3.38) |
| Poverty | 1.26 | (0.77 - 2.07) | 9.87** | (7.14 - 13.63) | 3.04** | (1.90 - 4.86) | 3.75** | (2.49 - 5.65) | 2.42** | (1.90 - 3.08) |
| Low maternal education | 0.86 | (0.60 - 1.23) | 3.58** | (2.51 - 5.12) | 0.91 | (0.59 - 1.40) | 0.75 | (0.48 - 1.16) | 0.90 | (0.73 - 1.11) |
| No parent working | 0.57* | (0.36 - 0.91) | 2.10** | (1.60 - 2.75) | 2.92** | (1.73 - 4.91) | 4.67** | (3.18 - 6.88) | 2.48** | (1.94 - 3.17) |
| Mother born outside UK | 27.41** | (18.88 - 39.79) | 42.91** | (31.23 - 58.94) | 5.19** | (2.76 - 9.76) | 68.78** | (41.16 - 114.93) | 11.00** | (8.31 - 14.57) |
| Christian | 0.22** | (0.07 - 0.69) | 0.02** | (0.00 - 0.12) | 1.78** | (1.18 - 2.70) | 10.03** | (4.92 - 20.43) | 0.79 | (0.61 - 1.02) |
| Muslim | 699.27** | (292.56 - 1,671.34) | 9,765.59** | (3,843.54 - 24,812.21) | 13.01** | (3.36 - 50.32) | 1,562.26** | (670.50 - 3,640.08) | 120.51** | (62.71 - 231.60) |
| Hindu and Sikh | 17,596.62** | (5,170.82 - 59,882.37) | 564.83** | (142.74 - 2,234.99) | - | - | - | - | 308.38** | (100.25 - 948.64) |
| Other | 21.66** | (7.54 - 62.26) | 11.00** | (2.39 - 50.56) | 0.72 | (0.10 - 4.98) | 6.77 | (0.82 - 55.64) | 4.25** | (1.92 - 9.41) |
| Single parent home | 0.37** | (0.21 - 0.64) | 0.54** | (0.39 - 0.74) | 5.03** | (2.97 - 8.52) | 3.69** | (2.40 - 5.66) | 2.41** | (1.92 - 3.03) |
| Low housing quality | 1.38 | (0.98 - 1.94) | 3.61** | (2.63 - 4.96) | 4.04** | (2.51 - 6.50) | 5.55** | (3.24 - 9.50) | 2.05** | (1.55 - 2.72) |
| ** p<0.01, * p<0.05 |  |  |  |  |  |  |  |  |  |  |

| Table S6. Univariate associations between ethnic group and parental and supervision variables | | | | | | | | | | |
| --- | --- | --- | --- | --- | --- | --- | --- | --- | --- | --- |
|  | **Indian** | | **Pakistani or**  **Bangladeshi** | | **Black**  **Caribbean** | | **Black**  **African** | | **Other ethnic**  **group** | |
|  | **OR** | **95% CI** | **OR** | **95% CI** | **OR** | **95% CI** | **OR** | **95% CI** | **OR** | **95% CI** |
| Maternal age 11-19 | 0.34** | (0.15 - 0.74) | 1.28 | (0.79 - 2.08) | 0.63 | (0.29 - 1.36) | 0.31* | (0.10 - 0.96) | 1.08 | (0.76 - 1.54) |
| 20-29 | 1.48** | (1.14 - 1.93) | 3.04** | (2.41 - 3.82) | 0.89 | (0.58 - 1.37) | 0.66 | (0.44 - 1.00) | 0.94 | (0.77 - 1.14) |
| 40+ | 0.26* | (0.10 - 0.73) | 0.91 | (0.45 - 1.82) | 3.06* | (1.28 - 7.30) | 3.27** | (1.97 - 5.43) | 1.10 | (0.57 - 2.14) |
| Grandparental co-residence | 15.59** | (11.25 - 21.59) | 8.78** | (6.85 - 11.24) | 1.36 | (0.47 - 3.94) | 1.00 | (0.49 - 2.06) | 2.06** | (1.31 - 3.24) |
| Another adult resident | 6.15** | (3.88 - 9.76) | 6.86** | (4.95 - 9.52) | 1.17 | (0.39 - 3.47) | 3.35** | (1.81 - 6.19) | 2.64** | (1.49 - 4.65) |
| High chaos | 0.40** | (0.28 - 0.56) | 0.58** | (0.46 - 0.73) | 0.64* | (0.43 - 0.97) | 0.64** | (0.48 - 0.85) | 0.79* | (0.63 - 0.98) |
| Smoking in home | 0.35** | (0.26 - 0.48) | 0.55** | (0.44 - 0.70) | 0.86 | (0.61 - 1.22) | 0.22** | (0.12 - 0.41) | 1.04 | (0.83 - 1.29) |
| Maternal frequent alcohol use | 0.12** | (0.07 - 0.20) | 0.02** | (0.01 - 0.03) | 0.56* | (0.34 - 0.93) | 0.06** | (0.03 - 0.12) | 0.53** | (0.41 - 0.68) |
| One sibling | 0.91 | (0.66 - 1.25) | 1.11 | (0.85 - 1.43) | 1.10 | (0.61 - 1.98) | 0.67 | (0.42 - 1.07) | 0.73* | (0.57 - 0.94) |
| Two or more siblings | 1.10 | (0.80 - 1.51) | 3.38** | (2.60 - 4.41) | 1.52 | (0.81 - 2.85) | 2.13** | (1.39 - 3.28) | 0.94 | (0.71 - 1.25) |
| Maternal no time spent with friends | 2.62** | (1.94 - 3.54) | 2.18** | (1.73 - 2.73) | 2.06** | (1.42 - 2.99) | 1.41 | (0.99 - 2.02) | 1.32* | (1.02 - 1.72) |
| Maternal no or low contact with parents | 4.92** | (3.47 - 6.97) | 3.50** | (2.82 - 4.35) | 1.94** | (1.28 - 2.96) | 11.77** | (8.03 - 17.27) | 2.88** | (2.32 - 3.57) |
| Poor area to bring up children | 0.84 | (0.55 - 1.30) | 1.87** | (1.30 - 2.67) | 2.78** | (1.65 - 4.70) | 2.15** | (1.27 - 3.65) | 2.02** | (1.47 - 2.78) |
| Low satisfaction with local area | 0.85 | (0.53 - 1.35) | 1.35 | (0.87 - 2.10) | 2.11* | (1.16 - 3.85) | 1.65* | (1.05 - 2.60) | 1.82** | (1.35 - 2.46) |
| Low social capital (composite of previous 4 vars) | 2.80** | (1.98 - 3.95) | 2.84** | (2.22 - 3.65) | 3.05** | (1.91 - 4.87) | 4.60** | (3.35 - 6.33) | 2.42** | (1.82 - 3.21) |
| ** p<0.01, * p<0.05 |  |  |  |  |  |  |  |  |  |  |

| Table S7. Univariate associations between ethnic group and values and parenting attitudes | | | | | | | | | | |
| --- | --- | --- | --- | --- | --- | --- | --- | --- | --- | --- |
|  | **Indian** | | **Pakistani or**  **Bangladeshi** | | **Black**  **Caribbean** | | **Black**  **African** | | **Other ethnic**  **group** | |
|  | **OR** | **95% CI** | **OR** | **95% CI** | **OR** | **95% CI** | **OR** | **95% CI** | **OR** | **95% CI** |
| Values to instil - independence | 0.13** | (0.04 - 0.42) | 0.09** | (0.04 - 0.18) | 475,470.70** | (9,064.05 - 24941646.54) | 0.15** | (0.06 - 0.34) | 0.42 | (0.12 - 1.48) |
| Obedience and respect | 0.58 | (0.58 - 0.58) | 1.52 | (1.52 - 1.52) | 316,113.90 | (316,113.90 - 316,113.90) | 3.36 | (3.36 - 3.36) | 0.45 | (0.45 - 0.45) |
| Art of negotiation | 0.48 | (0.21 - 1.11) | 0.55* | (0.33 - 0.92) | 0.69 | (0.21 - 2.19) | 0.88 | (0.24 - 3.22) | 0.55* | (0.33 - 0.91) |
| Respect for elders | 2,197,860.87 | (2,197,860.87 - 2,197,860.87) | 2,197,860.79 | (2,197,860.79 - 2,197,860.79) | 0.44 | (0.44 - 0.44) | 2,197,860.70 | (2,197,860.70 - 2,197,860.70) | 0.69 | (0.69 - 0.69) |
| Doing well at school | 1.28 | (1.28 - 1.28) | 2,116,982.99 | (2,116,982.99 - 2,116,982.99) | 2,116,982.95 | (2,116,982.95 - 2,116,982.95) | 2,116,983.02 | (2,116,983.02 - 2,116,983.02) | 3.78 | (3.78 - 3.78) |
| Religious values | 10.03** | (4.54 - 22.16) | 23.20** | (12.81 - 42.01) | 4.45** | (2.83 - 6.98) | 20.50** | (9.64 - 43.60) | 1.99** | (1.50 - 2.64) |
| Maternal negative attitude to working mothers | 1.24 | (0.79 - 1.94) | 4.91** | (3.90 - 6.19) | 1.32 | (0.69 - 2.51) | 1.92** | (1.19 - 3.11) | 1.62** | (1.21 - 2.18) |
| Unstructured parenting style | 1.01 | (0.73 - 1.39) | 1.42** | (1.15 - 1.75) | 0.57* | (0.33 - 0.98) | 0.75 | (0.55 - 1.03) | 0.96 | (0.76 - 1.21) |
| Any screentime | 0.89 | (0.22 - 3.67) | 0.29** | (0.20 - 0.42) | 0.64 | (0.15 - 2.83) | 1.21 | (0.29 - 5.03) | 1.08 | (0.42 - 2.79) |
| Irregular bedtime | 0.69 | (0.37 - 1.29) | 1.10 | (0.82 - 1.48) | 1.87* | (1.06 - 3.28) | 1.86* | (1.04 - 3.31) | 1.32 | (0.92 - 1.88) |
| ** p<0.01, * p<0.05 |  |  |  |  |  |  |  |  |  |  |

| Table S8. Proportion of children injured by ethnic group and stratified by maternal migrant status | | | | | | | | | | | | | | | | | | | |
| --- | --- | --- | --- | --- | --- | --- | --- | --- | --- | --- | --- | --- | --- | --- | --- | --- | --- | --- | --- |
|  | Full sample | | | | |  |  | UK born mothers | | | | | | Mothers born outside UK | | | | | |
|  | Total | No injury | | Any injury | |  |  | No injury | | Any injury | |  |  | No injury | | Any injury | | Compared to  white UK mothers | |
|  | N | N | *%* | N | *%* | OR | 95% CI | N | *%* | N | *%* | OR | 95% CI | N | *%* | N | *%* | OR | 95% CI |
| Total | 12717 | 9269 | *72.9* | 3448 | *27.1* |  |  | 8089 | *72.1* | 3129 | *27.9* |  |  | 1180 | *78.7* | 319 | *21.3* |  |  |
| White | 10904 | 7854 | *72.0* | 3050 | *28.0* | 1.00 | - | 7502 | *72.0* | 2921 | *28.0* | 1.00 | - | 352 | *73.2* | 129 | *26.8* | 0.79 | 0.46-1.33 |
| Indian | 297 | 230 | *77.4* | 67 | *22.6* | 0.73 | 0.53-1.01 | 100 | *78.7* | 27 | *21.3* | 0.70 | 0.44-1.13 | 130 | *76.5* | 40 | *23.5* | 0.79 | 0.46-1.33 |
| Pakistani or Bangladeshi | 664 | 514 | *77.4* | 150 | *22.6* | 0.80* | 0.66-0.98 | 151 | *70.1* | 63 | *29.4* | 1.17 | 0.90-1.52 | 363 | *80.7* | 87 | *19.3* | 0.68* | 0.48-0.96 |
| Black Caribbean | 134 | 101 | *75.4* | 33 | *24.6* | 0.97 | 0.65-1.46 | 73 | *70.9* | 30 | *29.1* | 1.20 | 0.76-1.90 | 28 | *90.3* | 3 | *9.7* | 0.29 | 0.08-1.11 |
| Black African | 134 | 174 | *84.1* | 33 | *15.9* | 0.46** | 0.29-0.74 | 33 | *86.8* | 5 | *13.2* | 0.37 | 0.13-1.09 | 141 | *83.4* | 28 | *16.6* | 0.51** | 0.31-0.85 |
| Other | 511 | 396 | *77.5* | 115 | *22.5* | 0.65** | 0.51-0.82 | 230 | *73.5* | 83 | *26.5* | 0.78 | 0.59-1.05 | 166 | *83.8* | 32 | *16.2* | 0.42** | 0.26-0.69 |
| ** p<0.01, *p<0.05 |  |  |  |  |  |  |  |  |  |  |  |  |  |  |  |  |  |  |  |

| Table S9. Adjusted odds for unintentional childhood injuries adjusted for sex socioeconomic factors, ethnicity and migrant status, and parent and household factors | | | | | | | | |
| --- | --- | --- | --- | --- | --- | --- | --- | --- |
|  | Adjusted for sex and socioeconomic factors | |  | Ethnicity and mother’s migrant status | |  | Adjusted for parental and household factors | |
|  | OR | 95% CI |  | OR | 95% CI |  | OR | 95% CI |
| **Sex** |  |  |  |  |  |  |  |  |
| Female | 1.00 | - |  | 1.00 | - |  | 1.00 | - |
| Male | 1.34** | 1.22-1.46 |  | 1.34** | 1.22-1.47 |  | 1.33** | 1.21-1.46 |
| **OECD Equivalised Income** |  |  |  |  |  |  |  |  |
| Above 60% median income | 1.00 | - |  | 1.00 | - |  | 1.00 | - |
| Below 60% median income | 1.09 | 0.97-1.21 |  | 1.14* | 1.00-1.28 |  | 1.07 | 0.95-1.21 |
| **Housing tenure** |  |  |  |  |  |  |  |  |
| Owner-occupied | 1.00 | - |  | 1.00 | - |  | 1.00 | - |
| Local authority | 1.18* | 1.03-1.35 |  | 1.20* | 1.04-1.38 |  | 1.12 | 0.96-1.30 |
| Housing association | 1.18 | 0.98-1.41 |  | 1.20 | 1.00-1.44 |  | 1.12 | 0.93-1.36 |
| Private rent | 1.22* | 1.01-1.47 |  | 1.21* | 1.00-1.46 |  | 1.15 | 0.95-1.40 |
| **Ethnic group** |  |  |  |  |  |  |  |  |
| White |  |  |  | 1.00 | - |  | 1.00 | - |
| Indian |  |  |  | 0.82 | 0.59-1.15 |  | 0.83 | 0.59-1.17 |
| Pakistani or Bangladeshi |  |  |  | 0.85 | 0.66-1.08 |  | 0.85 | 0.66-1.10 |
| Black Caribbean |  |  |  | 0.90 | 0.60-1.33 |  | 0.92 | 0.62-1.35 |
| Black African |  |  |  | 0.46** | 0.28-0.77 |  | 0.48** | 0.29-0.81 |
| Other |  |  |  | 0.64** | 0.50-0.81 |  | 0.64** | 0.50-0.82 |
| **Mother born in UK** |  |  |  |  |  |  |  |  |
| Yes |  |  |  | 1.00 | - |  | 1.00 | - |
| No |  |  |  | 0.83 | 0.69-1.00 |  | 0.85 | 0.70-1.03 |
| **Maternal age** |  |  |  |  |  |  |  |  |
| 30-39 |  |  |  |  |  |  | 1.00 | - |
| 11-19 |  |  |  |  |  |  | 1.05 | 0.88-1.25 |
| 20-29 |  |  |  |  |  |  | 1.17** | 1.05-1.30 |
| 40+ |  |  |  |  |  |  | 0.99 | 0.72-1.35 |
| **Household chaos** |  |  |  |  |  |  |  |  |
| Least chaotic |  |  |  |  |  |  | 1.00 | - |
| Most chaotic |  |  |  |  |  |  | 1.22** | 1.10-1.35 |
| **Any smoking in the home** |  |  |  |  |  |  |  |  |
| No |  |  |  |  |  |  | 1.00 | - |
| Yes |  |  |  |  |  |  | 0.98 | 0.89-1.08 |
| **Household Composition** |  |  |  |  |  |  |  |  |
| Two parent household |  |  |  |  |  |  | 1.00 | - |
| One parent household |  |  |  |  |  |  | 1.13 | 0.96-1.33 |
| *** p<0.01, * p<0.05, n = 12,717* | | | | | | | | |
